# The impact of fishing on a highly vulnerable ecosystem, the case of Juan Fernández Ridge ecosystem

**DOI:** 10.1371/journal.pone.0212485

**Published:** 2019-02-22

**Authors:** Javier Porobic, Elizabeth A. Fulton, Carolina Parada, Stewart Frusher, Billy Ernst, Pablo Manríquez

**Affiliations:** 1 Quantitative Marine Science Program, Institute for Marine and Antarctic Studies, Hobart, Tasmania, Australia; 2 CSIRO Oceans and Atmosphere, Hobart, Australia; 3 Institute for Marine and Antarctic Studies, University of Tasmania, Tasmania, Australia; 4 Centre for Marine Socioecology, University of Tasmania, Tasmania, Australia; 5 Departamento de Geofísica, Universidad de Concepción, Concepción, Chile; 6 Instituto Milenio de Oceanografía, Universidad de Concepción, Concepción, Chile; 7 Millennium Nucleus of Ecology and Sustainable Management of Oceanic Islands (ESMOI), Departamento Biología Marina, Universidad Católica del Norte, Coquimbo, Chile; 8 Departamento de Oceanografía, Universidad de Concepción, Concepción, Chile; Swedish University of Agricultural Sciences and Swedish Institute for the Marine Environment, University of Gothenburg, SWEDEN

## Abstract

The Juan Fernández Ridge (JFRE) is a vulnerable marine ecosystem (VME) located off the coast of central Chile formed by the Juan Fernández Archipelago and a group of seamounts. This ecosystem has unique biological and oceanographic features, characterized by: small geographical units, high degree of endemism with a high degree of connectivity within the system. Two fleets have historically operated in this system: a long term coastal artisanal fishery associated with the Islands, focused mainly on lobster, and a mainland based industrial demersal finfish fishery operating on the seamounts which is currently considered overexploited. The management of these fisheries has been based on a classical single-species approach to determine output controls (industrial fleet) and a mixed management system with formal and informal components (artisanal fleet). There has been growing interest in increasing the exploitation of fisheries, and modernization of the fishing fleet already operating in the JFRE. Under this scenario of increased levels of fishing exploitation and the high level of interrelation of species it might be necessary to understand the impact of these fisheries from a holistic perspective based on a ecosystem-based modeling approach. To address these challenges we developed an Atlantis end-to-end model was configured for this ecosystem. The implemented model has a high degree of skill in representing the observed trends and fluctuations of the JFRE. The model shows that the industrial fishing has a localized impact and the artisanal fisheries have a relatively low impact on the ecosystem, mainly via the lobster fishery. The model indicates that the depletion of large sized lobster has leads to an increase in the population of sea urchins. Although this increase is not sufficient, as yet, to cause substantial flow-on effects to other groups, caution is advised in case extra pressure leads the ecosystem towards a regime shift.

## Introduction

All human activities in the oceans and along coasts (e.g. tourism, fisheries, shipping, infrastructure) have some degree of impact on the ecosystems and no location has escaped from the human footprint [1]. One of the oldest known use of the oceans, fishing, can generate major disruptions in ecosystems such as decline of fish population abundance [2], changes in the physical structure of the environment [3], reduction of megafauna and top predators by bycatch [4, 5] and changes in the nutrient flow by discarding [6]. The likelihood and rate of recovery of an ecosystem to these disturbances (short-term or chronic) define its vulnerability [7], which can be related to both structural (physical structure such as coral on submerged edges and seamounts) and/or biological (e.g. maturation of fish at old ages) aspects of ecosystems. A vulnerable marine ecosystem (VME) is one that is easily disturbed but with a slow propensity to recover [7].

Seamounts are typical examples of VME, due to features such as small spatial extent, ecological complexity, hosting of vulnerable and endemic species (i.e. deep-sea coral; [7, 8]). However, the topographical and biophysical characteristics of these ecosystems suggest they can be more productive compared with the oceans that surround them [9]. Also, these seamounts have a great biodiversity of organisms: such as deep-sea corals, invertebrates, and fishes, many of them endemic [9, 10]. In addition, some of these species have great economic value, which has led to the development of lucrative fisheries surpassing more than 2 million tonnes of catch globally from these ecosystems since the 1960s [11]. However, many of these fisheries have not been sustainable and are currently classified as either over-exploited or depleted [11–14]. Seamounts can be classified as deep or shallow based on whether they approach surface waters. The type of fishing gear needed to fish a seamount is also typically influenced by its depth and distance from shore:

- Deep sea seamounts: Do not extend above 100 m sub-surface and are normally remote. Require more sophisticated ocean-going vessels to reach these seamounts and gears such as deep sea long lines or trawls are needed to fish them.
- Shallow seamounts: These are seamounts that reach to waters less than 100m sub-surface or break the surface to form islands (some of these are of sufficient size to support human settlements). These seamounts tend to have coastal fringes (typically with steep shelf profiles) and can support less sophisticated fishing activities with simpler vessels and fishing gears, such as shallow trawling.

The impact of fishing on VMEs has widely studied in the past [9, 11–13, 15] and has emphasized the negative impact generated by industrial trawl fisheries on such ecosystems [11, 12, 16], which can be in stark contrast to the low impact of some artisanal fisheries on these ecosystems [12]. The impact of any fishery on VMEs can be divided into direct and indirect effects [16].

The direct effects of fishing include mortality on target and non-target species (bycatch) and the physical impacts caused by the gear on benthic organisms and on the seabed [9, 16]. In its most negative aspects, these direct effects can lead to over-fishing, loss of biodiversity, degradation of suitable habitat [16] and fisheries-induced evolution [17].

The indirect effects of fisheries on ecosystems occur in several ways, which depend on the type of fishery and its operation [16]. This type of effect includes: i) impacts mediated by biological interactions between species in the ecosystem (e.g. competition and predation); ii) effect of discarding and offal on the contribution of nutrients to the ecosystem; and iii) the effect of ghost fishing (discarded, lost, or abandoned, fishing gear in the marine environment that continues to fish and trap animals) [16]. These 3 pathways fully influence the complex interaction networks of the ecosystems. Indeed, these modifications may have a cascade of second or third order effects and can reshape ecosystem structure, function and biodiversity [16]. For example, fishing can generate a change in the local species composition, which modifies the available habitat that in turn affects the species that use that habitat and consequently their predators [18].

The assessment of ecosystem effects of fishing (direct and indirect) is a difficult task, especially when there are multiple fisheries -artisanal and industrial- active around the one ecosystem. Determining the best management strategies in such a situation is quite challenging. Consequently, improved understanding of the relative effects from different forms of fishing is necessary for effective fisheries management and biodiversity conservation. Such knowledge is crucial for VMEs, given the uncertainties about their resilience [19].

**Fig 1:**
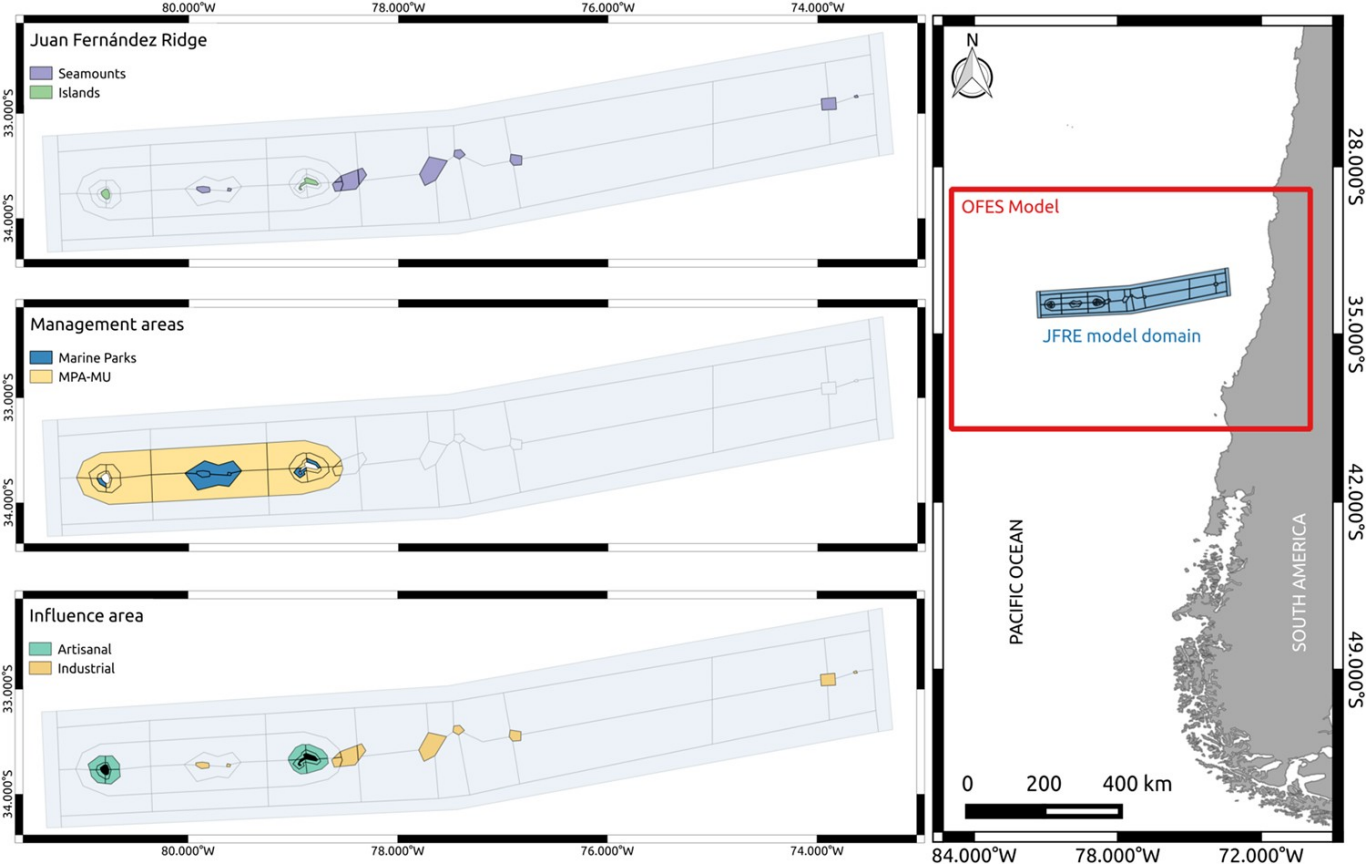
Atlantis JFRE model domain. The red square represents the area of coverage of the hydrodynamic OFES model subset. The maps in the left represent the characteristics that were considered for the division of the polygons: The geographic structure of the ridge (Islands and seamounts); Management area (Marine parks and Marine protected areas with multiple uses; MPA-MU); and areas of influence of fishing activity (Industrial and artisanal fleets).

The Juan Fernández Ridge Ecosystem (JFRE) meets all the criteria to be classified as a VME [8]. JFRE is an aseismic chain of seamounts and islands located off the coast of central Chile between 32.81° and 33.81°S, with a total length of approximately 800 km [20]. The JFRE is constituted by the Juan Fernández Archipelago (JFA) and a group of seamounts that include Friday and Domingo seamounts at the western edge and the O’Higgins guyot, close to the Chilean coast [20]. The JFA is located approximately 360 nm off Valparaiso and is formed by the Robinson Crusoe and Santa Clara Islands subsystem (RC-SC) and the Alejandro Selkirk Island (AS) located 90 nm to the west of RC-SC (Fig 1).

JFRE has unique biological and oceanographic features, characterized by: i) small and discrete geographical units (islands and seamounts); ii) high degree of marine and terrestrial endemism [21–25]; and iii) presence of mesoscale and submesoscale oceanographic structures promoting a high degree of connectivity within and between other systems [26–28]. Because of their significance for biodiversity the islands were declared a National Park in 1935 and a Biosphere Reserve by UNESCO in 1977 (www.unesco.org). Also, in 2016 the Chilean government declared a multipurpose marine protected area and several marine parks around the islands and seamounts (Fig 1).

There are only two small human settlements within the JFRE: Rada de la Colonia (AS), a temporary fishing village, inhabited each year only during the fishing season (October to May), with about 54 inhabitants including fishers and their families; and Juan Bautista, a town located in RC-SC. Juan Bautista is the only permanent town in the JFRE, with almost 930 inhabitants, which can grow to exceed a thousand inhabitants in summer, due to tourism and return of young islanders studying on the mainland.

### The fisheries

The local economy of the JFRE is based almost exclusively on the extraction and marketing of marine resources (specially lobster), although, in recent years there has been an increase in tourism activity, focused mainly on the pristine landscape and high biodiversity of the area. Eco-tourism is now the second most important economic activity for this town, with marine activities being a large component [29].

Two commercial fishing fleets have historically operated in the JFRE: i) a long term coastal and traditional artisanal fishery associated with the islands, mainly targeting lobsters (*Jasus frontalis*; [30, 31]), Juan Fernández (J.F.) morwong (*Nemadactylus gayi*; [32]) and more recently golden crab (*Chaceon chilensis*; [33]); and ii) a mainland based industrial demersal finfish fishery operating on the seamounts, which targeted on two species: orange roughy (*Hoplostethus atlanticus*) and alfonsino (*Berix splendens*), both of these industrial fisheries are currently considered over-exploited and are closed. A highly migratory pelagic fish fishery also operates in this system (e.g. swordfish; [34]), but these were not considered given the focus of the model on demersal and seamounts species.

The management of these fisheries (industrial and artisanal) has been based on a classical single-species approach. The two industrial fisheries were managed with an output control system that allocated catch shares (quota) to operators. The artisanal fisheries have specific regulations depending on their target species:

1. Lobster fishery: has been managed through a dual management system, based on: formal rules that include, type “SSS” regulations (Sex, Season and Size; [35]; i.e. legal size, season closure, no egg-carrying females), a moratorium on entry of new participating boats and restriction of gear to traps (all other methods are banned); and an informal traditional sea-tenure system [36].
2. Golden crab fishery: has one formal regulation, it is closed to the entry of new fishermen; and an informal agreement that controls the minimum landing size, which is not mandatory.
3. J.F. morwong fishery: has no formal regulation (at present).

Over the last 10-15 years there has been a growing interest in the artisanal fleet in increasing the exploitation of fisheries, currently considered at very low levels of exploitation and modernization of the fishing fleet that already operates in the JFRE [32, 37, 38]. However, due to the vulnerability of the JFRE, the high level of interrelation of species and the potential for increased exploitation with modernization or new sectors, the following question arises: What is the effect of the existing fisheries on the functioning and structure of the JFRE?; and What would be the effect of increased fishing on the ecosystem?

### Evaluating ecosystem effects of fishing

#### End-to-end models

To address this question, a holistic and integrated perspective is needed. One approach is to integrate the management and assessment of fisheries under an ecosystem’s framework. In the last decade, mainly due to technological advances, new approaches and tools have been developed to explicitly consider the entire exploited marine ecosystem [39–41]. Multispecies models that focus on the fishery, its target species and their closest connections in the ecosystem (or their other main drivers) is one valid approach [41]. However, in this case it was judged insufficient given the spatial and temporal processes that structure the functioning of the ecosystem surrounding the JFRE (e.g. the effect of the eddies on the productivity, larval connectivity). End-to-end models attempt to incorporate all the physical and ecological components of the ecosystem (including the human components), integrating them at different spatial and temporal scales [39]. These tools provide insights into ecosystem functioning, as well as the impact of human activities [39]. One of the largest end-to-end modeling platforms operating under a spatial framework is the Atlantis model [39, 42]. This model has been used for nearly two decades and is regularly being modified and applied to address new questions related to ecosystem functioning, management strategies, climate-change impacts and monitoring [43]. All these attributes make Atlantis well suited to explore and understand the impact of fishing activity on the vulnerable JFRE. This study aims to evaluate the impact (direct and indirect) of different fisheries on the Juan Fernández Ridge ecosystem by implementing an End-to-End model for this region.

## Materials and methods

### Atlantis-model

Atlantis is a modelling framework which includes the main components of the ecosystem and was developed specifically for use within management strategy evaluations [40]. Therefore, it includes: i) a representation of each of the major biophysical and human components of the ecosystem [43]; ii) adaptive management processes (monitoring, assessment and management decision) [44]; and iii) socioeconomic processes and drivers for the human use and behavior [43]. Moreover, Atlantis presents a range of alternative model formulations, which can be tailored to the user’s preferences. Hence, Atlantis can be implemented with different levels of complexity, from a simple model with few interactions to a complicated model with multiple biological connections and a complex structure of fishing fleets [43]. A detailed description of the Atlantis model and all its options and equations can be found in the model’s manual [45–47].

### Model structure and parametrization

#### Areas

Atlantis is a three-dimensional spatially explicit framework which is based on irregular polygons that account for the spatial structuring of the dominant processes (e.g. physical, biological and human impact) [43]. Furthermore, the model has a horizontal stratification which can incorporate vertical migration or depth-based variation in physical and biological components. The JFRE model covers an area of approximately 97,166 km^2^. This area includes a wide range of habitats, ranging from soft sediments and shallow rocky reefs in the coastal zone to pelagic habitats in the open ocean, pelagic and deep water habitats in the offshore area and seamounts (Fig 1). The division into polygons for the JFRE model was based on the main hydrodynamic, biological and fishery management components of the area (Fig 1). Parsing followed four main criteria: i) vertical and horizontal spatial distribution of the species and functional groups; ii) spatial distribution of nutrients, chlorophyll, or phytoplankton, based on model and bibliographic information; iii) spatial distribution of fishing fleets that currently or previously operated in the JFRE (artisanal and industrial); and iv) management areas, such as marine protected areas (MPAs), marine parks and exclusive artisanal fishery areas. Each of these polygons can have up to eight depth strata, with divisions at 20, 50, 150, 250, 400, 650, 1000 and 4300 m. This stratification is based on the vertical distribution of the functional groups [48–51] and the operation of the fishing fleets [33, 52, 53].

#### Oceanography

In Atlantis the biophysical sub-model was coupled with outputs from a three-dimensional hydrodynamic model to provide the main flows (e.g. water, salt, nutrients and heat) for the ecosystem. The eddy-resolving Ocean General Circulation Model for the Earth Simulator (OFES) [54, 55] was used to provide the oceanographic driving for the Atlantis-JFRE and configured over the period 1950-2011. OFES is a quasi-global high-resolution ocean model configured on a horizontal grid of 0.1° with 54 vertical levels (Fig 1) and monthly temporal resolution.

The Adaptation of OFES output to Atlantis requires: i) integrating OFES flows to the respective depth layers for each face in the available polygons; ii) correction for hyper-diffusion within polygons (which involves dividing the orthogonal flows between two polygons by the length of the interaction face between those polygons); and iii) statistical representation of relative seasonal eddy strength, so that the effect of eddies on productivity and connectivity can be represented. This last step is particularly important because these oceanographic structures are known to be abundant and important for productivity and connectivity in JFRE [26, 56, 57], but the polygonal structure is too coarse to be explicitly eddy resolving (see Other forcing variables).

#### Functional groups

This model is configured with 31 functional groups of which 16 are groups structured by age and 15 are gross biomass pools. In addition, the model includes pools of carrion, labile and refractory detritus (Table 1). These functional groups are aggregated groups of species with similar patterns of trophic interaction (diets), life history, and management measures. The interaction between these functional groups is mainly based on trophic relationships which were built based on literature reviews and reinforced with database searches (e.g. FishBase [58]).

**Table 1:** Biological functional groups in the JFRE model and their main configuration. The initial biomass in metrics tonnes, the classification (**A**) under fisheries means active fishery, (**S**) means secondary fishery or bait fishery, (**C**) represents currently closed fisheries and (**B**) was used for species that are bycatch of other fisheries (J.F. abbreviation stand for Juan Fernández). More detailed descriptions of the functional groups can be found in the supplementary material.

Code	Functional Groups	Initial Biomass (tonnes)	Modeled as	Fishery	Recruitment Model	Seasonal distribution
**SPL**	Spiny lobster	3.1e+03	Age-Structured	Artisanal (A)	Beverton-Holt	Yes
**GCR**	Golden crab	3.3e+02	Age-Structured	Artisanal (A)	Beverton-Holt	No
**BRC**	J.F. morwong	5.0e+03	Age-Structured	Artisanal (A; S)	Beverton-Holt	Yes
**ANG**	Moray eels	6.9e+03	Age-Structured	Artisanal (S)	Beverton-Holt	Yes
**VID**	Yellowtail amberjack	4.4e+03	Age-Structured	Artisanal (A)	Beverton-Holt	No
**ALF**	Alfonsino	3.8e+04	Age-Structured	Industrial (C)	Beverton-Holt	No
**ORO**	Orange roughy	2.5e+04	Age-Structured	Industrial (C)	Beverton-Holt	No
**SPF**	Small pelagic fish	7.7e+03	Age-Structured	Artisanal (S)	Beverton-Holt	Yes
**LPF**	Large pelagic fish	9.3e+03	Age-Structured	Artisanal (A; S)	Beverton-Holt	Yes
**SBF**	Small benthic fish	1.0e+04	Age-Structured	Artisanal (S)	Beverton-Holt	Yes
**LBF**	Large benthic fish	8.7e+03	Age-Structured	Artisanal (S)	Beverton-Holt	Yes
**MPF**	Mesopelagic fish	1.2e+05	Age-Structured	-	Beverton-Holt	No
**OTA**	J.F. fur seal	1.1e+01	Age-Structured	-	Fixed offspring	No
**DOL**	Dolphins	7.4e+02	Age-Structured	-	Beverton-Holt	No
**BIR**	Sea Birds	3.1e+00	Age-Structured	-	Fixed offspring	No
**CHO**	Chondrichthyes	6.8e+02	Age-Structured	-	Fixed offspring	No
**OCT**	J.F. octopus	7.2e+01	Biomass Pool	Artisanal (A; B)		No
**SQD**	Squid	1.3e+02	Biomass Pool	-		No
**SUR**	Sea Urchin	5.6e+03	Biomass Pool	Bycatch		No
**MOL**	Mollusc	1.4e+04	Biomass Pool	-		No
**SCR**	Other crustacean	1.4e+04	Biomass Pool	Bycatch		No
**COR**	Deep sea coral	5.9e+04	Biomass Pool	Bycatch		No
**SZO**	Small Zooplankton	5.2e+05	Biomass Pool	-		No
**MZO**	Medium Zooplankton	1.2e+06	Biomass Pool	-		No
**LZO**	Large Zooplankton	6.4e+05	Biomass Pool	-		No
**BFF**	Deposit feeders	2.0e+04	Biomass Pool	Bycatch		No
**LPH**	Large phytoplankton	1.6e+06	Biomass Pool	-		No
**SPH**	Small phytoplankton	1.9e+06	Biomass Pool	-		No
**MA**	Macroalgae	5.9e+04	Biomass Pool	-		No
**PB**	Pelagic Bacteria	2.6e+04	Biomass Pool	-		No
**BB**	Sediment Bacteria	8.0e+03	Biomass Pool	-		No
**DL**	Labile detritus	8.0e+03	Biomass Pool	-		No
**DR**	Refractory detritus	8.0e+00	Biomass Pool	-		No
**DC**	Carrion	8.0e+00	Biomass Pool	-		No

#### Other forcing variables

**Fig 2:**
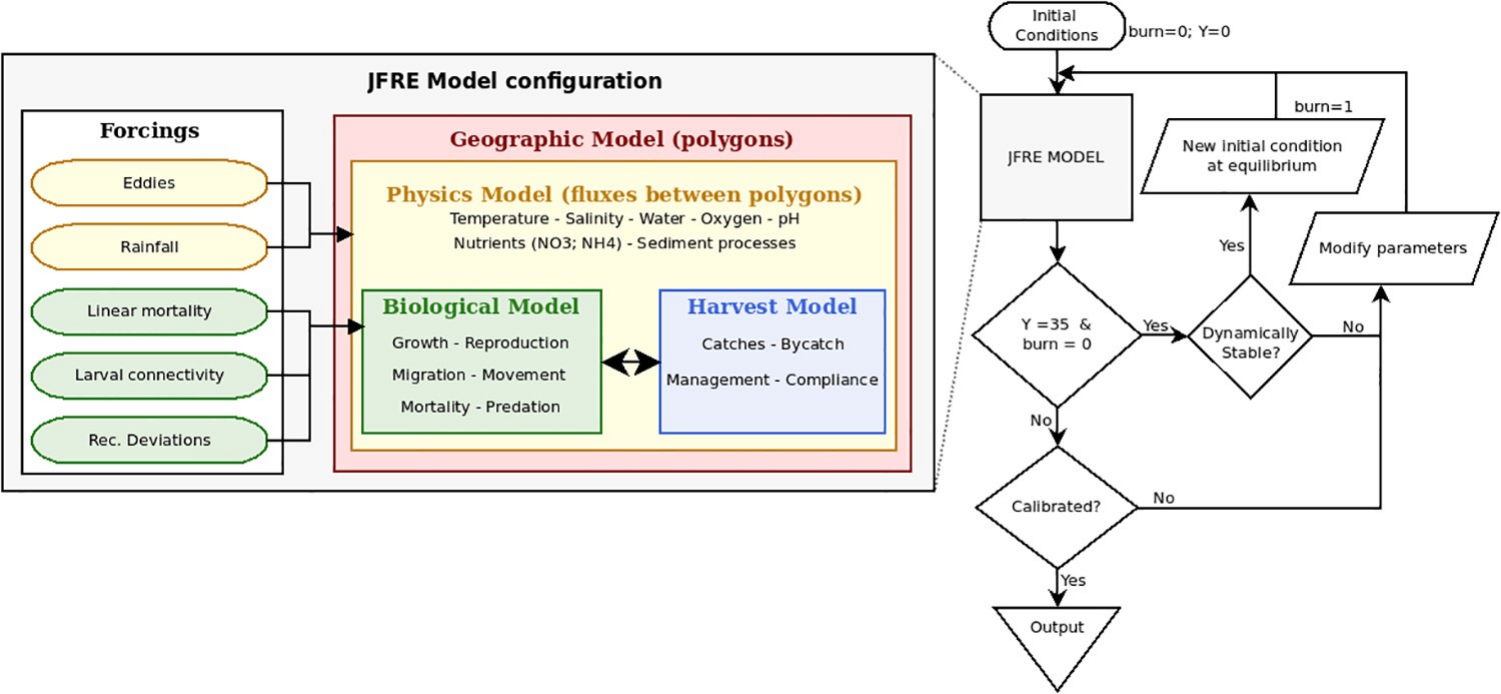
The left side includes the main components (biological, physical and economic) and forcings that were considered for the JFRE model. The flowchart shows that the model runs for 35 years if after this period the model is dynamically stable it creates a new initial condition at equilibrium. When the model is already dynamically stable, the calibration process is performed to represent the conditions observed in the ecosystem.

The JFRE model is supplied with temporal and spatial information from two different external forcings (Fig 2):

- **Eddies**: Due to the relevance of these physical processes to the JFRE, an analysis of the spatial distribution, time duration and seasonal variation of the eddies from the OFES model was done using the methodology described by Parada et al. [59].
- **Larval connectivity**: A coupled biophysical model for each age-structured functional group was used to establish the spatial structure of the recruitment and larval connectivity. This coupled model joins an oceanographic model (OFES model) and an individual-based model (IBM) using the Ichthyop simulation tool developed by Lett et al. [60]. The information used for the configuration of the IBM was: i) average date of spawning, based on the date of carrying of spawning eggs. For functional groups with more than one species, the average date of spawning between the different species was used; ii) area of release, based on the distribution of the adults or egg bearing females (for spiny lobster); and iii) average duration of the larval stage for each functional group (more details S11 Fig and S7 Table). The resulting larval dispersal matrices were read into Atlantis as a spatial forcing time series which condition box-to-box larval flow, as well as losses from the system. Further details on its parameterization and general pattern of results is provided in the supplementary materials.
- **Recruitment deviations**: For the spiny lobster functional group, a time series of recruitment deviation was used. This series extends from 1900 to 2015 and was obtained from the catch-at-length model by Porobic [61] (more details S9 Fig).
- **Rainfall**: The time series of nutrient contribution to the ecosystem (mainly NO_3_) was based on rainfall time series reconstruction around the JFRE islands [62]. The average value of nutrients contributed by effluent run-off from the island was extrapolated from literature [63] (more details S10 Fig).
- **Linear mortality**: To represent processes outside the scope of the model, a time series of linear mortality (where a simple mortality rate was imposed on the population with that rate itself trending linearly through time) was applied on both adult and juvenile of fur seal in the model. In the absence of more complete information on harvesting of pinnipeds in the region, this mortality time series was used to recreate the processes of depletion (and near extinction) experienced by this functional group (and to allow for the correct post-release dynamics). For the rest of the functional groups, a constant linear natural mortality rate was applied (to reflect non-predation mortality sources) with that rate obtained iteratively during the calibration process (see the section on the calibration process below)

#### Main biological processes

Atlantis includes several biological and ecological processes [46] which are tailored to match the system being modelled. The JFRE model incorporated the following processes:

1. **Seasonal Migration**: Three functional groups (dolphins, yellowtail amberjack and sea birds) migrate in and out of the model domain.
2. **Spatial distribution and Seasonal variation**: Spatial distribution (proportion by area) and its seasonal variation (if appropriate) were used (Table 1; more details S2–S8 Figs and S3–S5 Tables).
3. **Diel Vertical migration**: The pattern of the diurnal vertical migration through the watercolumn was used for zooplankton (large, medium and small) and for invertebrates such as squid and octopus and for fishes with not strict demersal or pelagic distribution.
4. **Recruitment and larval connectivity**: Recruitments were based primarily as a variant of the Beverton-Holt model or a fixed number of offspring per reproducing adult (Table 1). The spatial distribution of the recruitment (larval connectivity) was based on a biophysical model (as described above; more details S11 Fig and S7 Table).
5. **Temperature effect on biological processes**: The main processes affected by temperature in the JFRE model were physiochemical nutrient cycling, physiological rates, reproduction output, survival and spatial distribution. Details regarding the formulation of these processes can be found in the Atlantis manual [46]. The parameterization of these processes for the Juan Fernandez was based on available literature on the tolerances of species in the system (e.g. the range of temperatures they are observed to inhabit) and from metabolic and ecological studies on similar species from other systems.

#### Fisheries

The JFRE model included five different fisheries:

1. Three artisanal fisheries, which operate in RC-SC and AS. These fisheries include the Juan Fernández rock lobster, golden crab and J.F. morwong fisheries. Traps were used as fishing gear for the two crustacean fisheries, and longlines for the finfish.
  In addition, a two stage bait fishery is associated with the crustacean fisheries. The first stage involves catching small pelagic fish, which serve as bait for the second stage. In the second stage, the fishery catches moray eels, J.F. morwong and small and large benthic fishes which are then used as bait in the crustacean fishery [30], (see Table 1 for associated functional group codes).
2. Two industrial trawling fisheries targeting alfonsino and orange roughy, on seamounts.

For the purposes of this study a fishing mortality rate approach was used. The same rate was applied in all spatial cells occupied by the fished species and zoned open to fishing, but size specific selectivity was included via the use of a logistic length-based selectivity curve [47]. These fishing mortality and selectivity parameters were obtained from a number of technical reports available for fisheries in the region [26, 33, 64–67]. Bycatch from the industrial and artisanal fisheries was also incorporated in the model as a proportion of the total catch [47]. Bycatch included coral, other crustaceans, molluscs, sea urchins, deposit filter feeders and octopus for the artisanal fleet; and sharks, coral, other crustaceans and deposit filter feeders for the industrial fleet [68, 69].

#### Model calibration and metrics

The calibration of the model aims to reproduce the observed system trends and the patterns observed in the ecosystem such as catches and abundance of the functional groups. This model calibration was performed in two stages (Fig 2):

1. Bringing the model to equilibrium: The biomasses of the functional groups were taken to a state of equilibrium avoiding the extinction or overgrowth of any one group. This included a spin-up simulation of 31 years under a constant fishing pressure with all biomasses remaining within a factor of 2 of their initial biomasses. Subsequently, the biomass and size structures at the end of the simulation were used as the initial condition of the model for all subsequent runs. The hindcast of the model started in 1950.
2. Fine calibration: A refined calibration based on a pattern oriented approach [70] was used to adjust the outputs of the model to the patterns and time series observed in the ecosystem (i.e. catches and biomass).

A model skill assessment was carried out to evaluate the performance of the model to reflect the observed trend and magnitude of the observational data for the JFRE. Model efficiency, Reliability index and Pearson correlation between the estimated and observed landings was used to test the model performance as proposed by Olsen et al [71]. Only the catches of spiny lobster, orange roughy, alfonsino and the abundance of fur seal were used in this assessment as these were the only time series available.

#### Scenarios and simulations

A set of scenarios were implemented to assess the direct and indirect effects of fishing on the JFRE (Tables 2 and 3). These scenarios aim to evaluate:

1. *The historical effect of fishing*: To understand the effect of existing fisheries on the functioning and structure of the JFRE we simulated scenarios with historical fishing mortality for the industrial and artisanal fleets (and the combine effects of both). The output of these scenarios was compared against a simulated unfished ecosystem (Table 2).
2. *The future increase in fishing effort for the artisanal fisheries*: As there is no as yet agreed level of increase proposed for the fisheries of the JFRE, a range of plausible scenarios for the expansion of the artisanal fishery (given patterns of development elsewhere in Chile) were trialed. In the first simulation we projected the model 35 years into the future using a constant fishing mortality (at 2016 level). In the subsequent simulations, fishing mortality levels were increased (above the reference level in 2016) by 50% for crustacean fisheries (spiny lobster and golden crab; ICRUS50%); or by 50% for both fisheries (IBOTH50%); or by 100% just in the finfish fisheries (IFISH100%; Table 3). We acknowledge that these are fairly simplistic scenarios, but we were primarily interested in gross effects not nuance, given that those directly involved in the fishery are yet to define more detailed scenarios. In addition, the lobster and golden crab fisheries use the same fishing gear, the same type of boats and in some instances the same boat fishes both resources in a single fishing trip. Thus, including a scenario where the changes in these fisheries was matched seemed to be a logical inclusion.

Note that it is assumed in these scenarios that the industrial fishery remains closed. This assumption was made as no research has been carried out to evaluate stock conditions, suggesting that there is no clear intention from the Chilean government to reopen these over-exploited fisheries. Furthermore, the seamounts that sustained a large part of the industrial fishing effort have been declared as national parks or marine protected areas and thus are unlikely to re-open to legal fishing. For simplicity in these initial scenario assessments we have chosen not to include potential illegal fishing activities.

**Table 2:** Scenarios used to evaluate the effects of historical fishing activity in the JFRE.

Scenario	Fishing mortality	Fleet
Unfished	No Fishing	-
Historical Artisanal	Historical level	Artisanal
Historical Industrial	Historical level	Industrial
Historical JFRE	Historical level	Both

**Table 3:** Scenarios used to evaluate the effects of future fishing activity in the JFRE. **C** stands for current level of fishing pressure, **I** is the increase in fishing pressure and **I**+ is the cumulative increase of fishing pressure for both types of artisanal fisheries.

[1cm]								
Scenario	Crustacean		Finfish					
	SPL	GCR	BRC	VID	SPF	LPF	SBF	LBF
Business as usual (BAU)	C	C	C	C	C	C	C	C
ICRUS50%	I	I	I	I	I	I	I	I
IBOTH50%	I	I	I+	I+	I+	I+	I+	I+
IFISH100%	C	C	C	I	I	I	I	I

#### Software

The development and simulation of the JFRE was performed using Atlantis-trunk 6178 model [72]. This version of Atlantis was compiled in gcc 4.8 [73] under the operating system Ubuntu 14.04 LTS [74]. For pre and post-processing R software version 3.2.3 was used [75]. All these programs were executed under GNU Emacs 24.3.1 [76]. All versions of the parameter files and R codes used during the development of this work were stored in GitHub [77] and are available in (https://github.com/jporobicg/Atlantis_JFRE_Model). The Atlantis code can be accessed from the CSIRO SVN repository after registering with the Atlantis user group (https://research.csiro.au/atlantis/). To simulate the larval connectivity of the age class groups the Ichthyop simulation tool version 3.3 [78] was used.

## Results

### Metrics and description

**Fig 3:**
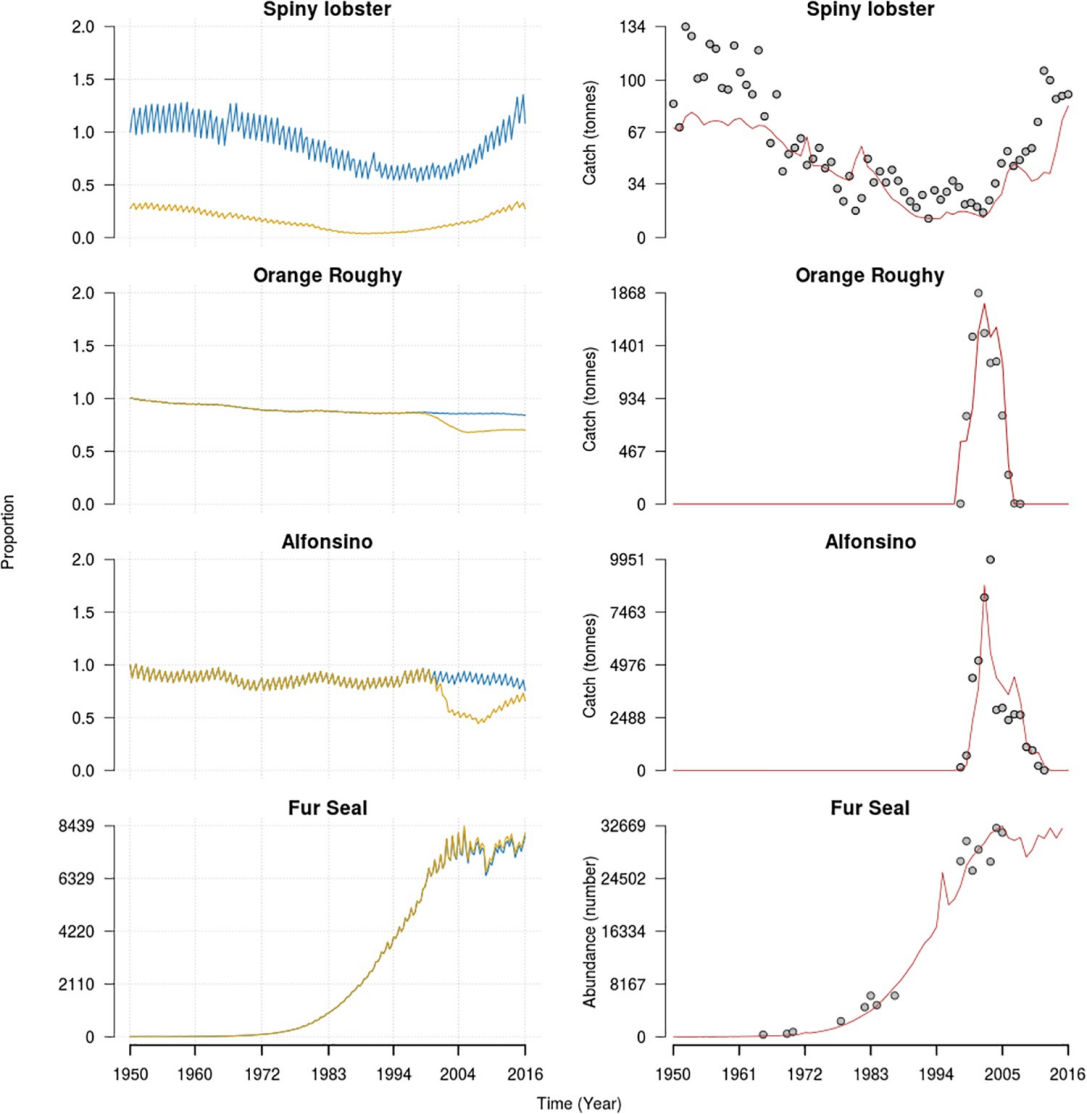
In the left column are presented the time series of the biomass relative to the initial biomass of an unfished (blue line) and fished (yellow line) ecosystem. The first three rows of the right column represents the time series of simulated catches in Atlantis (red line) and observed (gray dots). For the Fur seal, the dots represented the observed abundance and the red line is the estimated abundance from Atlantis.

The JFRE Atlantis model had a spin-up period of 31 years, where all the functional groups reached a dynamic stability. This spin-up duration was based on the time that the orange roughy population required to reach a stable size structure and a stable dynamic state. On average, the other functional groups required 10 years (+/-3) to reach this state. The JFRE Atlantis model (hindcast mode) covered the period from 1950 to 2011 to match the time span of the oceanographic model. During this period, most of the functional groups remained within the limits of established dynamic variation (+/-50% of the initial biomass). The model reproduced a marked intra-annual biomass variability coupled with inter-annual variations (more details in S12 Fig). The model skill assessment for all the functional groups, using all available information shows a strong and significant similarity between the simulated and observed time series of catches and abundances (Fig 3; Table 4). In addition, all the analyzed time series had model efficiency coefficient values close to 1, which implies a high symmetry and match between predictions and observations (Table 4). In addition, the reliability index values show low average values of the deviation factor between the observed values and the predictions.

**Table 4:** Metrics used to determine the skill in model assessment. p-values are provided in brackets.

Metrics	Alfonsino	Orange roughy	Spiny lobster	Fur seal
Correlation (Spearman)	0.88 (< 2.2*e* − 16)	0.87 (4*e* − 4)	0.83 (< 2.2*e* − 16)	0.95 (< 2.2*e* − 16)
Reliability index	4.06	6.69	1.52	1.58
Model Efficiency (ME)	0.999	0.999	0.999	1

**Fig 4:**
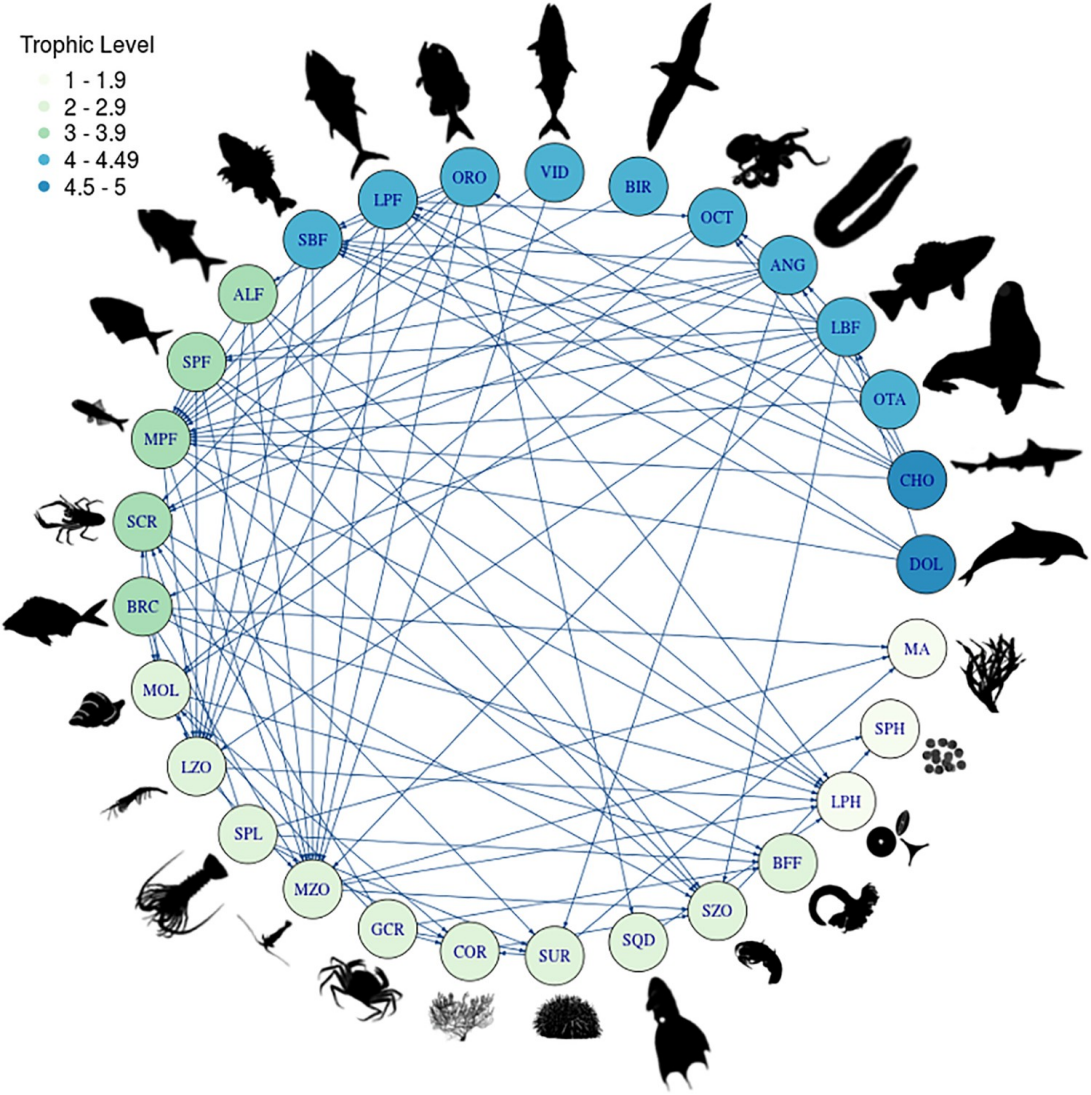
Food web of the Juan Fernández Ridge ecosystem. The code representing the functional groups can be found in Table 1.

The structure of the trophic web simulated by Atlantis shows levels ranging from 1 for primary producers (e.g. macroalgae) to 5 for top predators (e.g. mammals and sharks; Fig 4). There is a high concentration of functional groups at trophic levels 2 to 3 characterized by crustaceans and molluscs and at trophic levels 4 to 5 which is composed by finfish, mammals, birds and sharks (Fig 4). Based on the number of trophic connections, the main prey of this ecosystem are plankton groups (zooplankton and phytoplankton) and the mesopelagic fish.

### Historical effect of fishing (hindcast)

**Fig 5:**
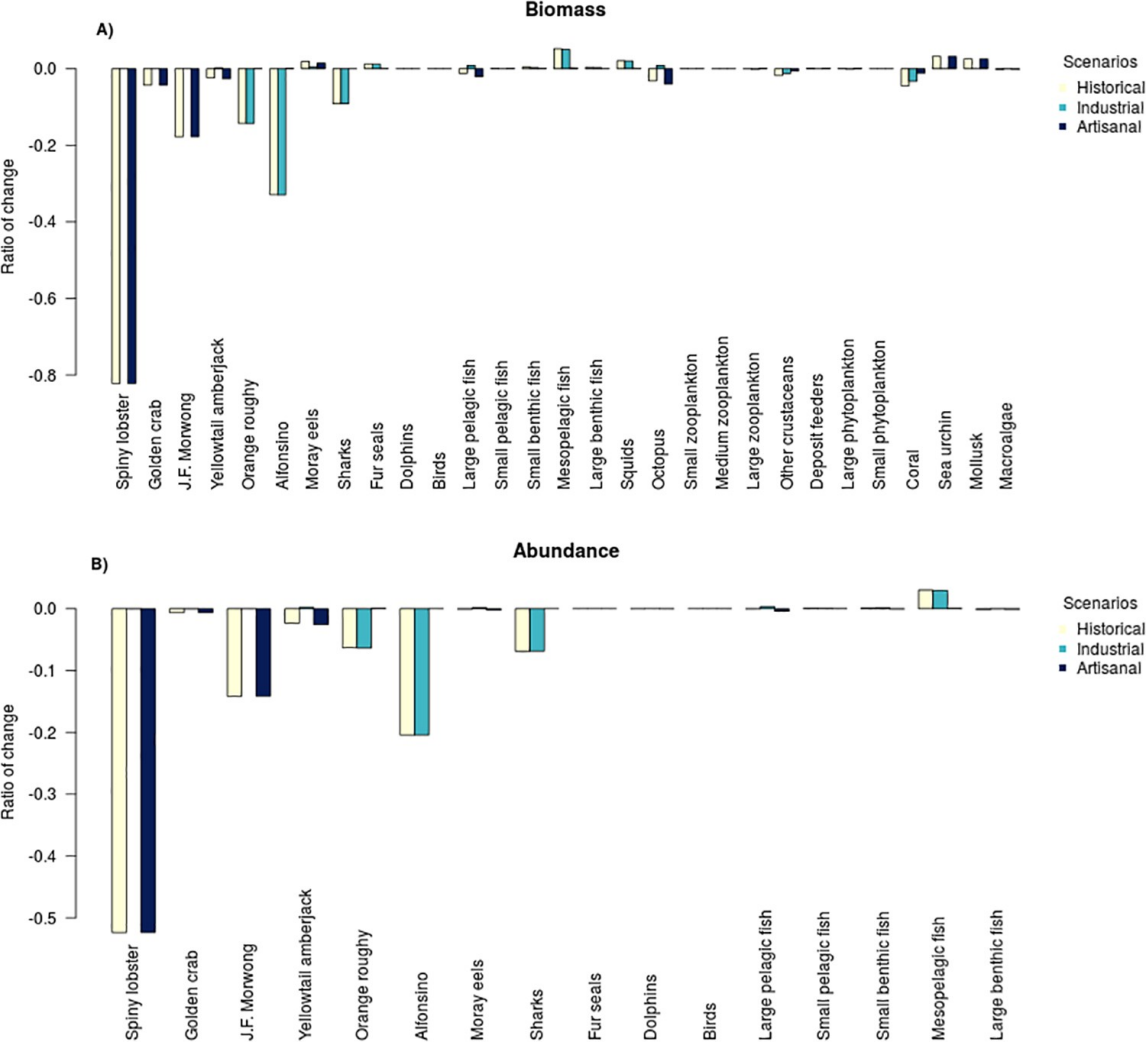
Relative change in biomass (A) and abundance (B) for the scenarios with only artisanal, industrial and the historical fisheries (industrial + artisanal). An unfished ecosystem is the base case for comparisons. Note that the y-axes is the ratio of change against the starting conditions—so a -0.5 result indicates a 50% decrease and a 0.5 result indicates a 50% increase -.

Most functional groups have a strong similarity between the simulated unfished and fished ecosystems (see S12 Fig), with the exception of spiny lobster, orange roughy and alfonsino which show the strongest divergences between those scenarios (Fig 3). There is not a strong synergy or antagonism between the effect of both fleets (artisanal and industrial). The interaction between them was a small (less than 1%) antagonistic effect, which is observed for yellowtail amberjack, octopus and large pelagic fishes (Fig 5).

#### Artisanal fisheries

The simulations show that the lobster stock has undergone a total biomass depletion of approximately 81% compared to the unfished system (Figs 3 and 5A); although in terms of abundance the drop is 53% (Fig 5B). Golden crab presents a similar pattern between abundance and biomass, where the proportional difference in simulated biomass is 4% and observed abundance is less than 1% (Fig 5). Juan Fernández morwong shows a reduction in almost 11% of its biomass compared to an unfished ecosystem. The overall effect of artisanal fishing, with the exception of golden crab and spiny lobster, is reflected discreetly in a few functional groups: a decrease of 4% in the abundance of octopus; less than 3% drop for yellowtail amberjack; less than 1% decrease for other crustaceans (Fig 5A); and an increase around 3% in mollusc and sea urchin biomass; Moray eels have a small increase of 2% in biomass, which is not reflected in the abundance (which has a reduction of less than 1%).

**Fig 6:**
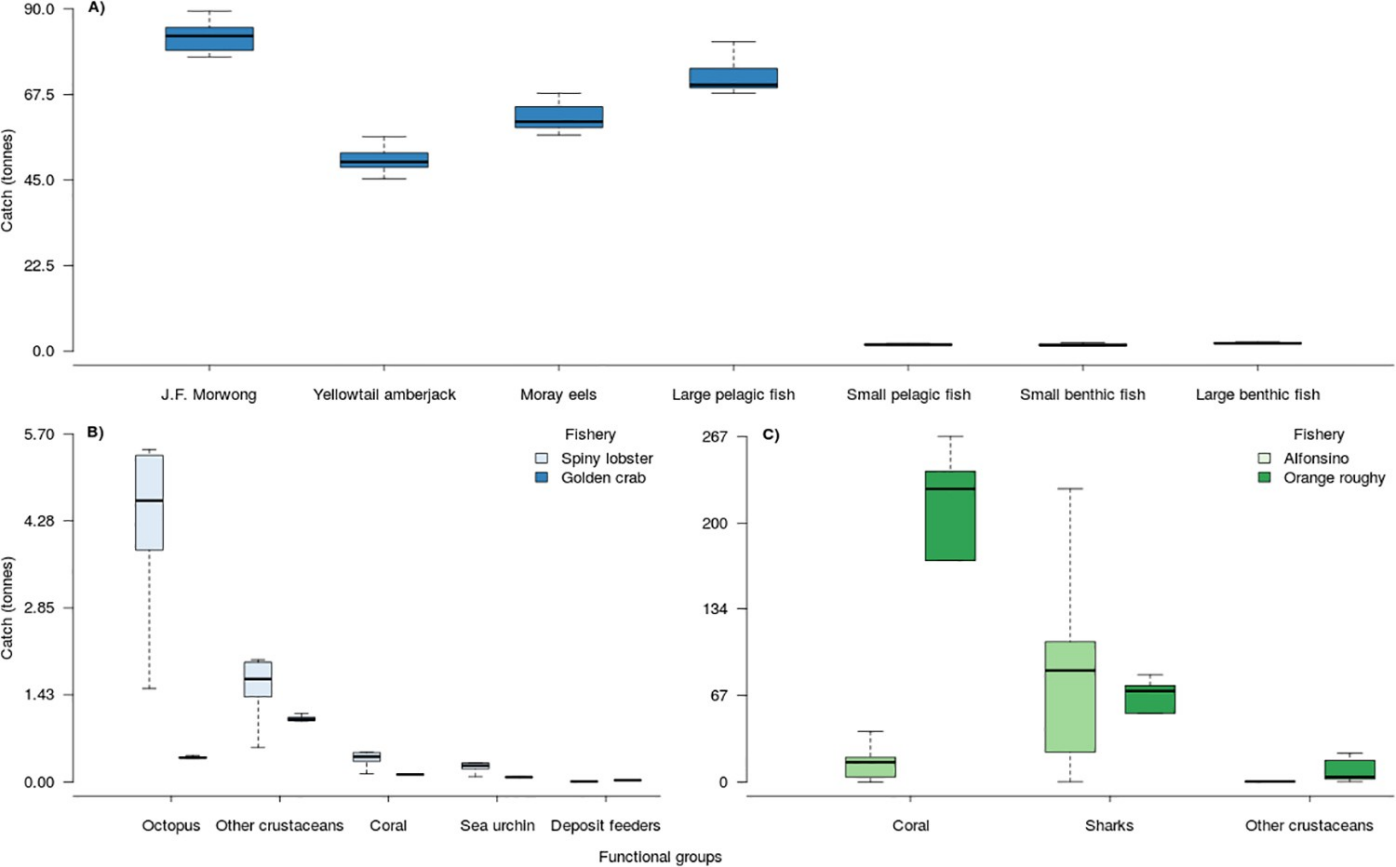
Summary of the catch distribution for the total bait caught from artisanal fisheries (A)), the total artisanal bycatch (B)) and the industrial bycatch (C)). The upper and lower limits of boxes represent the 25^th^ and the 75^th^ percentile and the middle line the 50^th^ percentile of the data distribution. The whiskers represent the range of the catches.

In the artisanal fleet, bait catches are focused on a few functional groups divided in two categories according to the level of fishing impact. First a high impact category composed of the catches of the functional groups: large pelagic fishes, J.F. morwong, moray eels and yellowtail amberjack with average annual catches of 84.5 (+/-5.5), 83.8 (+/-3.9), 63.4 (+/-3.2) and 51.3 (+/-2.9) tonnes respectively (Fig 6A). A second category less impacted is composed of small pelagic fishes, small benthic fishes and large benthic fishes that present annual historical catches close to 3 tonnes.

The spiny lobster fishery shows the highest levels of bycatch, mainly composed of octopus with an annual average of 5.18 (+/-2.7) tonnes and other crustaceans with 1.91 (+/-0.99) tonnes (Fig 6B). In addition, there is incidental catch of corals, sea urchins and deposit feeders with average catches of 0.46 (+/-0.24), 0.3 (+/-0.15) and 0.01 (+/-0.003) tonnes annually respectively (Fig 6B). For the golden crab fishery, other crustaceans are the main bycatch with an average of 1.08 (+/-0.03) tonnes annually (Fig 6B).

#### Industrial fisheries

During the fishing period, the population of alfonsino reached its highest catch after the biomass had been reduced by 50% with respect to its initial unfished biomass (Fig 3). After this depletion, a rapid recovery of its biomass is observed, reaching 85% of its biomass by the end of the hindcast simulation compared to an unfished scenario (Fig 3). In contrast orange roughy drove down the total biomass by almost 30% and the rate of recovery is slower, seeing it reach 83% of its total biomass by the end of the hindcast simulation(Fig 3). Compared with the unfished scenario, on average, the alfonsino biomass and abundance was reduced by 32% and 20% respectively. For orange roughy this reduction was around 15% for the total biomass and 4% for the abundance. The general effect of the industrial fishery is reflected in a few functional groups (Fig 5) with a positive effect on the biomass of mesopelagic fishes (almost 5%), squids (2%) and Fur seals (1%).

The bycatch in the industrial fleet is mainly composed of sharks, corals and other crustaceans (Fig 6C). This contributes to the predicted reduction of the biomass of sharks (of around 9%) and coral (around 2%; Fig 5a).

In the alfonsino fishery, shark catches reached an average 79.8 (+/-61) tonnes per year, which corresponds on average to 3.6% of the annual volume of alfonsino catches, corals make up 0.6% with an average of 14 (+/-10.7) tonnes and other crustaceans reached 0.74 (0.8) tonnes on average which make up 0.03% of the volume of catches of alfonsino (Fig 6C). For the orange roughy fishery, the coral bycatch reaches 193 (+/-85) tonnes per year, equivalent to 12.73% of the annual catches of this fishery. In the case of sharks these are equivalent to 3.95% of the orange roughy catches, with an annual average of 60.28 (+/-26.6) tonnes, and other crustaceans make up the equivalent of 0.46% of the annual orange roughy catches with an annual average of 8.43 (+/-8.9) tonnes of bycatch(Fig 6C).

### Projections

The comparison of the state in the projected scenarios (from the 1^st^ of January 2016 to January 1^st^ 2051) against the unfished ecosystem scenario are summarized briefly below.

#### Business as usual (BAU)

**Fig 7:**
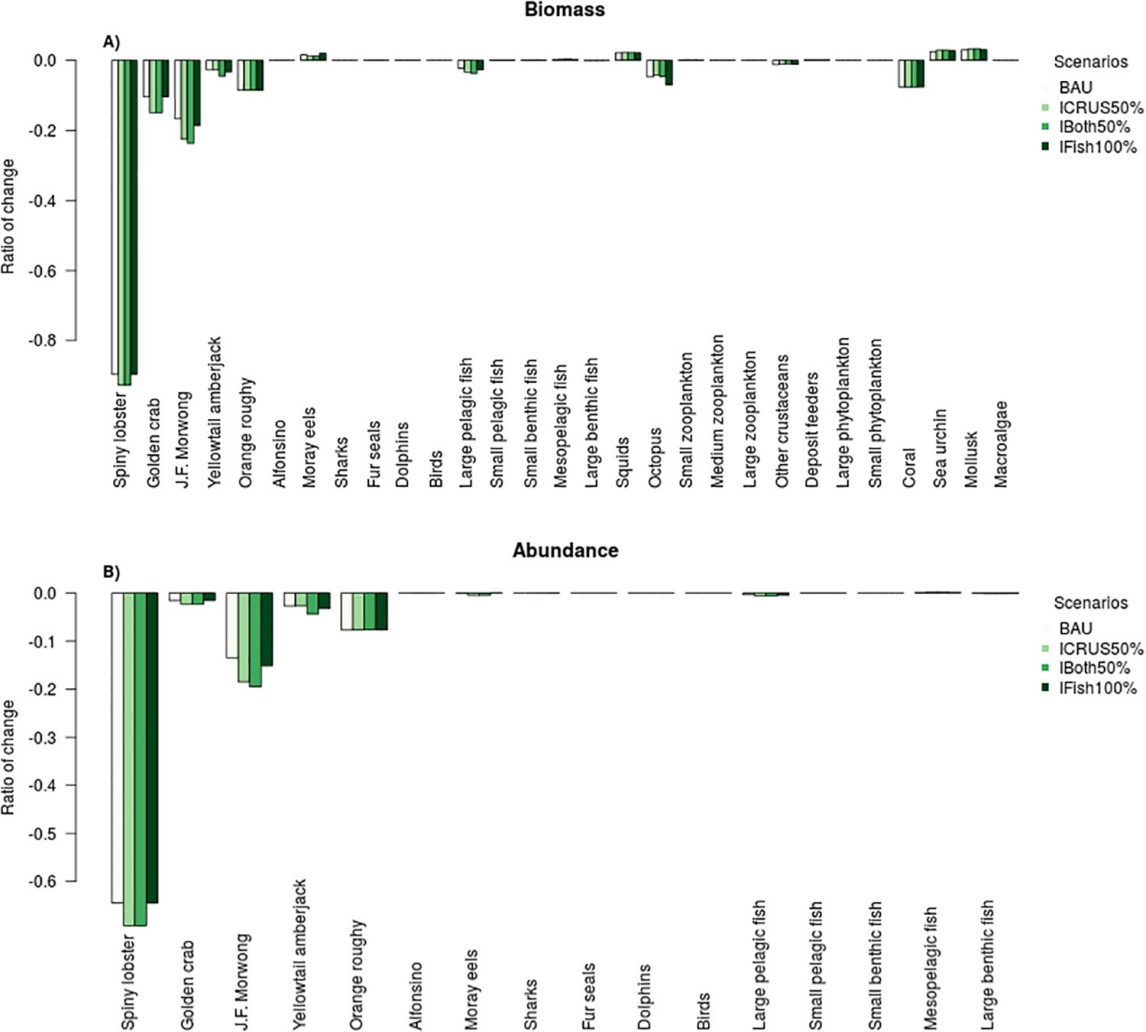
Relative change in biomass (A) and abundance (B) for the business as usual (BAU), a 50% increase in fishing effort of crustaceans (ICRUS50%), 50% increase in fishing effort for crustaceans and finfish (IBoth50%) and 100% increase in fishing effort for finfish (IFish100%). An unfished ecosystem is the base case for comparisons. Note that the y-axes is the ratio of change against the starting conditions—so a -0.5 result indicates a 50% decrease and a 0.5 result indicates a 50% increase -.

The BAU scenario shows similar results as the hindcast model for the artisanal fisheries (Figs 5 and 7). With a marked reduction of 87% and 63% of spiny lobster biomass and abundance respectively. In addition, there is a reduction of 10% of golden crab, 16% of J.F. morwong and 5% of octopus biomass. Moray eels, sea urchin and molluscs all increase by approximately 2%.

#### Increase fishing mortality of crustaceans (spiny lobster and golden crab)

In the projections, the increase in fishing mortality by 50% in the crustacean fishery shows a decrease of approximately 92% in biomass and 69% in abundance for spiny lobster for the last 5 years of simulation (Fig 7). For golden crab the decrease in biomass and abundance reaches 15% and 2% respectively (Fig 7). In finfish, the most evident effect is the decrease in J.F. morwong biomass of approximately 15% and 11% reduction in abundance compared to an unfished scenario (Fig 7). In addition there is an increase of around 1.8% in biomass of moray eels. Compared to the BAU scenario there is a decrease of 29% of biomass and 13% of the abundance of spiny lobster (Fig 7). In golden crab, this reduction was almost 5% for biomass and less than 1% in abundance. For finfish, compared with the BAU projection, the biomass and abundance of J.F. morwong decreased by 7% and 6% respectively (Fig 7). For all other functional groups there is almost no change (i.e. less than 1%) with respect to the BAU scenario.

**Fig 8:**
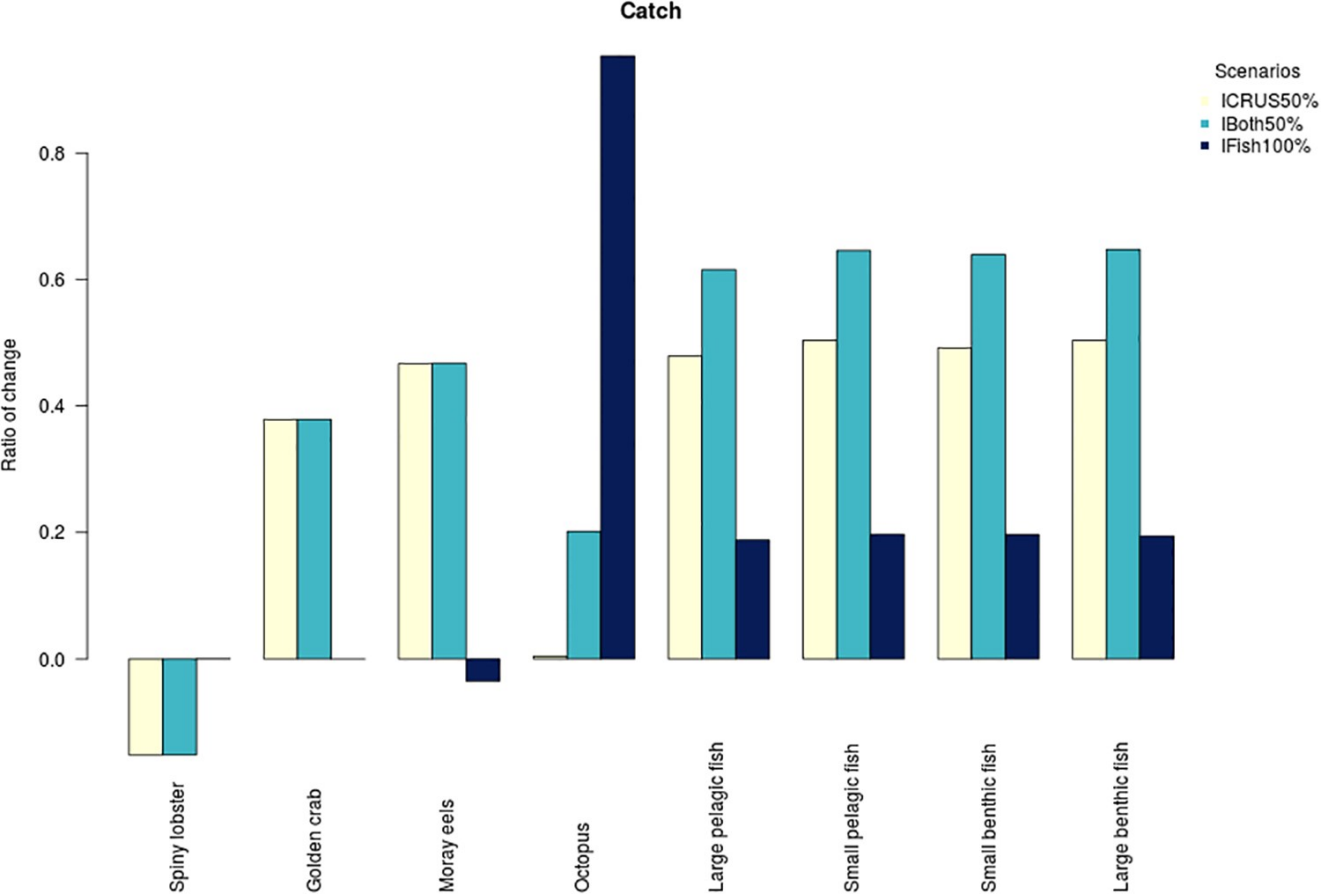
Relative change in catch for the last 20 years of projection for 50% increase in fishing effort of crustaceans (ICRUS50%), a 50% increase in fishing effort for crustaceans and finfish (IBoth50%) and 100% increase in fishing effort for finfish (IFish100%). The catches from a business as usual scenario is the base case for comparisons. Note that the y-axes is the ratio of change against the starting conditions—so a -0.5 result indicates a 50% decrease and a 0.5 result indicates a 50% increase -.

In terms of catches these increased by approximately 50% for all finfish and moray eels and by almost 40% for golden crab compared to the BAU projection (Fig 8). In the case of lobster, the total catch is reduced by around 15% compared to a BAU projection.

#### Increase fishing effort for finfish and crustacean artisanal fisheries

The results of this projection are similar to the scenario for the increase in crustacean fishing mortality, although there is a slight decrease in abundance and biomass for some functional groups such as J.F. morwong and octopus. Compared with the BAU scenario, the most important changes are the drop in biomass and abundance of spiny lobster, golden crab and J.F. morwong (Fig 7). For catches, they increase around 65% for finfish, 50% for moray eels, almost 40% for golden crab, when compared to the BAU projection. In contrast, for lobsters there is a greater than 15% reduction in final catch levels compared to a BAU projection.

#### Increase fishing effort in finfish artisanal fisheries

The effects of this scenario of increased fishing mortality in finfish fisheries are only reflected in a few functional groups. Octopus biomass declined by 7% and J.F. morwong declined by 18% (representing an 14% reduction in abundance). In addition, there was a small observed increase in the abundance and biomass of moray eels (around 2%). Catches increased around 20% for finfish and 95% for Octopus compared to the BAU projection. For the moray eels there is a reduction of 3% and there are almost no changes for spiny lobster and golden crab catches when compared to a BAU projection.

Despite a complete closure of the industrial fisheries in these projections, there was an 8% change in biomass (6% in abundance) for orange roughy and an increase of 2% for squid (Fig 7). Post 2024, there is no difference in projected biomass for alfonsino between a fished and unfished scenario.

## Discussion

The Juan Fernández ecosystem is a vulnerable marine ecosystem that has being impacted by industrial and artisanal fishing. Assessing the degree of impact of these fisheries on the ecosystem is vital as a source of information for fisheries management. It provides an indication of the need for management measures aimed at ensuring the sustainability of resources and the ecosystem [16, 79]. To face this challenge, an ecosystem model for JFRE was implemented using the Atlantis ecosystem framework. The implemented model can efficiently represent the responses and fluctuations of the ecosystem. It shows a high degree of synchronization between the observed and estimated catch and abundance, reproducing the main temporal trends and variations. Thus, under the current configuration and calibration, this model efficiently represents the JFRE biological and harvesting dynamics, which makes it a good tool to address questions about the ecosystem status, and dynamics.

### Trophic interactions

The functioning of JFRE, like other seamount ecosystems, is highly dependent on local primary production [80]. The major component of the food web is centered on phytoplankton, zooplankton and mesopelagic fishes, similar in structure to other deep-sea ecosystems [81]. In addition, there is a difference in the trophic structure between species that inhabit different depths. This apparent change in diet is mainly determined by the type of prey accessed by functional groups living at different depths in seamounts [9]. For example, deep water species such as orange roughy have a diet different from those associated to shallower waters such as Juan Fernández morwong (Fig 4). Moreover, it is likely that the same functional group will have differences in their diets depending on the depth of the seamount they inhabit [9], this is something that was not analyzed in detail in this model due to a lack of sufficient observational data. Such an analysis would however be useful as part of future work so as to increase understanding of how the potential existence of alternative trophic pathways may (or may not) influence the outcome of development and management scenarios in the modelled area.

### Artisanal fisheries

In the model, the lobster fishery has a strong impact on the spiny lobster population, reducing the biomass by 80%. Even with this large removal, only 50% of the abundance is extracted from the ecosystem. Two mechanisms can explain or contribute to this outcome:

1. The fishery is removing only the largest lobsters, leaving the smallest and most numerous ages in the ecosystem which can be observed in the age class size distribution [67].
2. There is an increase in survival of smaller lobster due to the reduction of intraspecific competition for shelter [82] or more available food due to the removal of large-sized lobsters.

Even though there is a strong total biomass reduction in the spiny lobster stock, this fishery does not show any sign of collapsing in the future. An important factor in explaining the sustainability of the fishery is the minimum legal size of lobster extraction [83], which is supported by this analysis. The minimum landing size of the fishery is 115 mm which is much higher than the average size of first maturity of 81.1 mm [84]. In this species, the average growth increment per year can vary between 3 to 6 mm [83] which allows the population to have between 5 to 11 reproductive events between first maturity and recruitment to the harvestable component of the population [84].

#### Ecosystem

Aside from the sharp decline in lobster biomass, the artisanal fishery has an small overall impact on the ecosystem. One reason for this is because, beyond the direct removal of the lobsters, the amount of biomass removed as bycatch or bait by the fishery is very small compared to the total estimated available biomass for the potentially affected functional groups.

In terms of predation, the decrease in lobster biomass did not have a strong impact on the ecosystem. The spiny lobster is an opportunist and generalist group with a wide spectrum of prey [85, 86], this means that the impact on the prey was not centered only on one item type, which means that the effect of a reduction in lobster biomass is dampened by dispersion over many groups. Species such as sea urchins and molluscs are the most influenced by the abundance and biomass decrease of lobsters (Fig 5). In terms of how a decline in spiny lobster biomass influences its predators, the model again suggests it does not have a major impact. According to the simulations this is because the lobster does not represent the main item of any species, and most of its predators are species that can feed on other functional groups. The species that feed on lobster (e.g., large benthic fish, moray eels, octopus and sharks) can focus their effort on capturing other functional groups that are more available when the abundance of lobster is low.

Given the strong depletion of the large sized spiny lobster due to fishing, a more marked growth of the populations of urchin was expected. The adult lobster has been reported as a key species controlling the abundance of sea urchin populations elsewhere [87]. Empirical observations in other systems indicates that top-down control of sea urchins by spiny lobster stops the overgrazing effect of the urchins on macroalgae. This mechanism decreases the probability of the creation of barrens of sea urchins avoiding extreme changes in the structure of the ecosystem [87]. Within the modelled system, although there is an increase in the biomass of sea urchins (3%; Fig 6), it is more moderate than expected, based on observations from other ecosystems [87, 88]. In JFRE, unlike other ecosystems, moray eels are also one of the main predators of sea urchin, controlling its abundance. Therefore, in the absence of spiny lobster that trophic role is being carried out by the moray eels and may be an ecosystem stabilizer (more details S13 and S14 Figs). The strength of this relationship remains to be verified in the real system, but if it is the case, then a greater decrease in eel abundance and biomass could result in major ecosystem change due to increased sea urchin populations and overgrazing of the macro-algal habitat as seen elsewhere [87, 88].

Another direct means in which fisheries can influence the JFRE is via the bait fishery. There is a two stage bait fishery associated with the capture of crustacean (lobster and golden crab) [89]. In the first stage capturing pelagic species that are used to catch larger species, which are later used as baits in the crab and lobster fisheries. In the model, this bait fishery is mainly associated with 4 functional groups: i) Large pelagic fishes, and which make up the first-order baits; ii) J.F. morwong; iii) the moray eels; and vi) yellowtail amberjack. These last three make up the baits used in the traps.

Despite the non-trivial levels of catches in the bait-fishery, the model does not report a depletion of the biomass in these functional groups due to the high total biomass levels for these groups. This means that the total bait catch levels are a small proportion of the total biomass for each of these abundant functional groups. The J.F. morwong is the only functional group that shows a biomass reduction, which is mainly explained by: 1) lower overall biomass in comparison to the other functional groups (partly because it is a single species whereas the other groups are composites of several species); 2) the depletion effect corresponds to the cumulative effect of both the bait fishery and the small direct fishery for J.F morwong.

In the case of the moray eel, the stability in the biomass masks a change in abundance. The model indicates that there are fewer but larger eels (i.e. less abundance but a slight increase in biomass). This increase in the biomass of moray eels likely results from the combined effect of:

1. An increase in prey biomass, due to the reduction of the predation effect of lobster there is an increase in the availability of several prey that are also part of the diet of moray eels (i.e. sea urchin, other crustaceans and mollusc). This increase in prey results in a larger food supply and therefore an increase in individual body mass, generating larger moray eels.
2. Reduction in intraspecific competition. The slight reduction in the moray eels abundance due to the bait fishery, reduces the intraspecific competition for food, increasing the effective availability of prey for moray eels.

The baits used for the spiny lobster and golden crab fisheries are composed of species of high trophic level (e.g. large pelagic fishes and yellowtail amberjack) that generally would not be part of the diet of these species or at least not in those quantities (Fig 5; [67]). This change in diet may have a seasonal impact increasing the trophic level of these functional groups, but under the current configuration of the model this is not being analyzed. This change in diet (i.e. amount and type of nutrients) would influence the survival and growth of lobster, most likely via a food subsidy [90, 91] but also with higher energy content prey becoming more available.

The small overall footprint of the bait fishery also masks spatio-temporal heterogeneity. The high level of spatial aggregation of fishing effort in a small area means there is still significant potential for a larger effect in that area [38, 90]. This is not implicitly represented in the model given its current configuration (uniform fishing mortality) and is something that should be added in future updates.

Other ways in which fisheries may potentially affect the JFRE is via bycatch. The levels of bycatch reported by the model are similar to those previously estimated for the artisanal fisheries of the archipelago [67]. While both the modelled crustacean fisheries have similar fishing gear (traps), there is a marked difference in the total amount of bycatch (Fig 7). This difference is mainly due to the bathymetric distribution of the species. The lobster fishery operates from 3 to 200 meters [89] and the golden crab fishery from approximately 300 to 600 meters [33]. This marked bathymetric division is reflected in the bycatch composition of each fishery, which is characterized by species associated with the respective bathymetric ranges [67].

Interestingly in both cases the amount of bycatch is not sufficient to cause significant or widespread effects; the bycatch of the artisanal fleet in the JFRE appears to affect very few functional groups and does not have an important overall impact on the ecosystem at its current levels.

We should, however, be cautious about this model-based bycatch finding. This is especially relevant for coral bycatch, which is composed of coral species that can live for hundreds of years and whose growth is extremely slow [92, 93]. The representation of these groups in the model was fairly rudimentary and is unlikely to have captured the true delays involved in this slow growth and maturity. In addition, the model does not differentiate between the two types of coral (deep and shallow), which could further mask the ecological effect of this removal. A more detailed analysis of this effect needs to model both corals separately. Therefore, the removal of corals could have a larger and longer term impact than what is reported by the model.

### Industrial fisheries

#### The fisheries

The two functional groups (i.e. orange roughy and alfonsino) captured by the industrial fleet have different population responses to the effect of fishing. Alfonsino showed a decrease in biomass and total abundance, whereas for orange roughy the total catch was just a small part of the total biomass of the population. In 2006, the orange roughy fishery was closed, arguing a population depletion based on the sharp fall in catches [94]. The model results suggest, that this reduction in catches is likely to be related to other factors affecting resource availability and not directly due to low biomass levels. What is observed in the model has been reported previously [65] and several hypotheses (apart from overfishing) have been proposed to explain low availability of orange roughy:

- Alteration of the reproductive behavior which leads to transitory problems in resources availability. The fishing effort on orange roughy was concentrated between May and August, but centered at spawning period (June-July) [95]. This direct and continuous impact of fishing (during consecutive fishing seasons) on the reproductive stock may have generated a change in the reproductive behavior of the species, which is reflected in an apparent decrease in abundance.
- Strong inter-annual availability. It has been reported that orange roughy presents strong interannual variations in its recruitment (or spawning success) [96]. This would generate strong interannual changes in the vulnerable biomass which would be reflected in a strong variation in catches. This hypothesis is not supported by the model, although interannual variation is observed in the recruitment, there are no strong variation in the biomass of older groups. However, caution needs to be shown since the reduced complexity of the larval processes and may underestimate true inter-annual variability.
- Changes and reduction of the suitable habitat for orange roughy as a result of the impact of fishing gear used by the industrial fleet. This alteration of the orange roughy habitat could then induce changes in the spatial distribution of the species. The seamounts impacted by fishing became less productive and therefore are avoided by orange roughy [97]. This process would leads to a localized stock depletion at scales below that being resolved in the model.

Although the model reports that the orange roughy resource is healthy and that the losses in the catches may have originated from other processes, without new information and the analysis of observations that are informative around the real world veracity of the alternative explanations, the hypothesis of overfishing must be maintained under the precautionary approach [65].

In terms of stock recovery, even though both fisheries were closed for several years (2006 for orange roughy and 2012 for alfonsino) their estimated biomass and abundance has not fully recovered. The recovery of alfonsino has been faster than orange roughy, reaching almost 85% of the unfished biomass by the end of 2016. The model projections, indicate that a full recovery of the alfonsino biomass will be achieved after 12 years of fishery closure. These values are similar to those proposed by other modeling exercises in which the estimated recovery period was found to be 13 years post the fishery being closed [66]. In orange roughy, the recovery of biomass has been at a much slower rate compared to alfonsino. The model suggests that the fishery is not likely to recover before 44 years of closure reaching about 92% recovery. This estimate is similar to the result by other modeling approaches [65] where stability is observed around 90% of the spawning biomass with respect to the virgin biomass. This slow rate of recovery of orange roughy is due to its life history characteristics (slow growing and late maturing).

#### The ecosystem

The industrial fleet has an overall low impact on the ecosystem and it is highly localized and concentrated on a few species (functional groups). Beyond the direct impact on the target species, the reduction of the abundance of alfonsino and orange roughy saw an increase in the biomass of mesopelagic fish. This functional group is an important component of the diet of orange roughy, constituting almost 50% of their diet [98], and alfonsino—where mesopelagic are the second most important prey item [99]. Therefore, a decrease in the abundance and biomass of these two species is rapidly reflected in mesopelagic fish biomass. In addition, this increase in biomass in the mesopelagic fishes has a direct effect on the biomass of fur seals which are the group’s main predator [100]. The model suggest that the exploitation of orange roughy and alfonsino has a positive effect on the recovery of the fur seal population. This means that there is the potential that as these fish stocks recover, there may be a negative impact on the fur seal populations. This outcome may result in either: 1) prey switching by the fur seals and potentially increased interactions with other fisheries; or 2) a decline in the population which may lead to conservation concerns or tourism impacts.

The main negative effect of the industrial fisheries on the ecosystem is on corals and shark (Fig 6). Even though the reduction of biomass due to bycatch is very small compared with their total biomass, the impact is highly localized. These bycatch species are sessile or with low mobility (benthic sharks) so the real effect of bycatch is below the scale of the model cells. Therefore, it would be difficult to capture in full detail the effects given the current geographical structure of the model. Due to the small spatial extent of the trawling footprint of these fisheries, the level of depletion and loss of species richness is highly localized and represents a small amount compared to the total biomass of these species [68]. To more thoroughly evaluate this localized effect of industrial bycatch, it is necessary to reconfigure the spatial structure of the model. For example, the current polygons could be subdivided using the areas where the fishing effort was more intense [68] or use a telescoping spatial approach [101]. A change like this in the spatial resolution of the model would have two important effects:

1. It would help determine the extent of any habitat depletion and loss of species richness at the local level, especially in the areas highly impacted by fishing.
2. It would facilitate an analysis of the magnitude of heterogeneity in ecosystem outcomes and the tracking of any localized shifts in bycatch levels and potential declines in affected species. For example, a local depletion of bycatch species, as the fishery develops and the richness and abundance of species decreases, could lead to a drop in the levels of bycatch [68, 102]. Such a mechanism would be important to understand as lowering bycatch levels are often interpreted as the positive outcome of fishery management actions (which in some cases they are, but it is important to distinguish the true underlying cause before using the index as a performance measure).

### Projections

The future (controlled) increase in fishing appears to be a viable option for all artisanal fisheries in this system, except for the lobster fishery where an increase is detrimental to the lobster population and its catches. For example, a 50% increase in fishing mortality for crustaceans over the current value sees the total removal of lobster dropping below the BAU scenario (Fig 8). This reduction can be linked to:

1. The current depletion of the oldest age classes of the population. The current size structure of the population in based on ages below the minimum landing size. This means, that even when there is an increase in fishing mortality, the overall increase in catch will not be significant. This finding is not in conflict with real world observations, where the size structure of the catches are centered on the minimum landing size with few individuals in the larger sizes [67]. Actually, it has been proposed that this is a “recruitment fishery”, where the catches are based on what recruits to the minimum legal size of the fishery each year [89].
2. The extra reduction in larger sizes results from the increase in fishing mortality which further diminishes the reproductive potential of the lobster. This reduction of reproductive potential will have a direct impact on the total population recruitment and therefore the future number of lobsters that will enter the fishery. This relationship between reproduction, biological recruitment and fisheries production has been previously demonstrated for other lobster species [103].

While scenarios explicitly exploring spatial dynamics in the system were not considered here, based on the spatial dynamics of the JFRE artisanal fisheries [38], it is likely that an increase in fishing effort and decrease in catch will have negative effects on fisheries management. The historical tenure of discrete fishing spots for each boat (owned by a fisher or family) has been part of the traditional management system and has been identified as the main controller of fishing effort [38, 67]. Therefore, if the fishing effort is increased (more boats or more fishing capacity), the need for increased fishing grounds will increase the probability of spatial overlap of the territorial footprint of different fisher’s fishing areas. This increase in the probability of overlapping fishing sites increases the risk of conflict, which could endanger the traditional cooperative management of this fishery. Considering all of the above, increasing the fishing effort in spiny lobster, does not seem the most appropriate approach, especially as it leads to:

1. a reduction in overall catch compared to the BAU scenario (Fig 8) and, given the increased effort assumed by the scenario, the reduced catch results in lower CPUE resulting in lower financial returns to the fisher and impacts on the local economy and community;
2. a potential increase in the risk of conflict between fishers, due to the spatial structure of the fishing fleet and the traditional spatial ownership of fishing areas;
3. a significant increase in the amount of bait used which is ultimately lost as waste or as food for other organisms (e.g., smaller lobsters, fish, other crustaceans)—as there is fewer crustaceans to catch even with the higher bait use—and therefore it would be better to use the fish in a more appropriate way (e.g. sell them at the market).

In the case of the golden crab fishery, this increase in fishing mortality enhances the catches and does not have a very strong impact on the biomass of this group. This means that the stock and the ecosystem could potentially support an increase in fishing mortality of golden crab with minimal apparent risk of having a major impact. Other assessments of this resource estimate that it is an underdeveloped fishery and there have been suggestions of further development of the fishery, as an alternative to the lobster fishery [64].

In the modelled artisanal finfish fishery, despite a 100% increase in the current fishing mortality rate the total reduction of biomass and the catches are lower than the increase of 50% fishing mortality for the crustacean fishery (Figs 7 and 8). This is because the main source of fishing mortality for finfish is associated with the crustacean fishery’s bait fishery [67]. Beyond the bait fishery other fishing pressure is minimal for finfish and therefore the total increase in fishing mortality imposed on finfish by the crustacean’s bait fishery is higher than the increment in the artisanal finfish fishery alone.

In terms of the impact of industrial fisheries, even though the fishery has been closed for several years, the biomass of orange roughy and coral are still below the unfished scenario at the end of the model run. This is because both functional groups continue to recover from the impact of fishing. This slow recovery was expected especially due to the slow growth of these functional groups, which makes it difficult to quickly return to levels of virgin biomass. Coral are species of very low resilience and ability to recover from this type of disturbance [92, 93].

### Future needs

JFRE is a remote vulnerable marine ecosystem far from the Chilean coast. This geographical distance has made the scientific research in this system scanty and irregular over time. The distance from the coast of Chile increases the costs and logistical constraints of any scientific research. Despite this, many research initiatives have been developed over several years but the majority have focused on fishing related scientific research. In the last few years this has gradually changed and new research including other aspects of the ecosystem has been developed. While, this is an improvement, more research is needed to achieve a solid understanding of the system and it’s sustainable management.

The development of this ecosystem model has answered several of the questions concerning the dynamics of the JFRE, but it has also posed many challenges for the future. If we seek to improve the understanding of the functioning of the ecosystem through a modeling tool such as Atlantis, there are many aspects that would benefit from further refinement, updates or additions (Table 5). While there are many areas of modeling that can be improved, there are priorities especially from the point of view of resource risk management, monetary cost and logistic capacity (Table 5).

**Table 5:** Information and model components that are needed for a future update or to use the model with data assimilation. These components are divided by: **Priority**, that means how urgent they are; **Type**, which include new model components to configure in the Atlantis framework (socio-economic components) and data which can be composed of single data or time series; **Periodicity**, correspond the ideal maximum time lag needed for the information; and **Spatial** if the information that is needed should be at the spatial level. Note that information at the level of the species should primary include key species from the ecosystem such as Spiny lobster, J.F. morwong, sea urchins.

Variable/Model	Priority	Type	Periodicity	Spatial	Comments
Diet	High	Data	seasonal	Yes	Reinforces knowledge about trophic relationships between functional groups.
Abundance	High	Data	3 years	Yes	Supports calibration, setting of initial conditions and model skill assessment.
Temperature	High	Data	1 month	Yes	Essential for evaluating effects on populations and for including future scenarios of climate change.
Socio-economic	High	New component	-	No	Pivotal for understanding the drivers of behavior and for translating management actions and outcomes through to consequences on the fishing community.
Primary production	High	Data	seasonal	Yes	Crucial in an ecosystem such as JFRE for understanding the supporting drivers of the system and for exploring cascade effects across functional groups.
Nutrients	Medium	Data	seasonal	Yes	Important for understanding the spatial and temporal variation of these variables, especially for understanding how production has changed in the past and may change into the future.
pH	Medium	Data	seasonal	Yes	Important for exploring the implications of ocean acidification.
Larval connectivity	Medium	Data	1 year	Yes	In an ecosystem like JFRE, the spatial connectivity can have a strong effect on the abundance and spatial distribution of functional groups (so understanding its past, current and future patterns is important).
Larval settlement	Medium	Data	seasonal	Yes	The larval settlement information can help to calibrate the temporal changes in abundance and help to understand the connectivity.
Fisheries Management	Medium	New component	-	-	This is fundamental to exploring new management alternatives suitable for this ecosystem, for reproducing past patterns of consequences and exploring the efficacy of current and proposed options and any associated challenges.
Tourism information	Medium	New component	-	-	Tourism is of growing economic importance on the islands and including its activities could have important effects on the JFRE ecosystem.

The collection of time series of different ecosystem components and other relevant indicators is also an important need. Having time series allows for an understanding of the temporal variations and trends of different aspects of the ecosystem (e.g., recruitment, abundance, temperature, nutrients, pH). The availability of time series in JFRE are scarce and only consist of some specific efforts related to specific fished resource species (i.e. Spiny lobster, golden crab) or several endanger species (e.g. Pink-footed shearwaters, fur seals). Although these time series are essential, it is necessary to reinforce them with others that describe other aspects of the ecosystem. Due to the human and institutional effort and the associated costs, these would likely need to be carried out on different time scales (months to years; Table 5). The exact form of such an effort would depend on the dynamics of each variable, the source of the data and the costs of obtaining the data. For example, algal biomass can be obtained at fine spatial and temporal scales from satellite imagery but requires a cost of obtaining the imagery and processing the data. Similarly, water temperate data can be obtain from sensors. In contrast, ecological components such as urchin numbers or macroalgal cover require trained personnel (e.g. divers) to undertake transects on an annual basis (Table 5).

## Conclusion

The JFRE Atlantis model has proven to be beneficial for describing the status of the JFRE commercial and artisanal fisheries. Also, providing insights into the impact on the ecosystem that would be expected to result from changes in fishing pressure (i.e. Artisanal fishery). Industrial fishing is located on the deep seamounts and restricted to larger trawler vessels based out of mainland Chile. Due to concerns about the level of depletion and the rate of recovery, these fisheries have been closed since 2006 for orange roughy and 2012 for alfonsino. This industrial fishing has a fairly localized impact on both target species and bycatch. In the case of alfonsino, it has an important direct effect with a high depletion of the biomass of this species. For the orange roughy stock, no significant depletion of the total biomass was observed. An explanation of this is that the observed drop in catches is due to other factors associated with availability such as alteration of reproductive behavior, reduction of available habitat or strong inter-annual variation of recruitment. Despite this low estimated reduction in total biomass, the recovery towards historical population sizes has been slow due to their life characteristics such as extremely slow growth. As for bycatch, both industrial fisheries present a high level of bycatch and this is associated with sessile or low mobility species such as coral and benthic sharks. Seamount fisheries are characterized by fine resolution intensive fishing activities. While the JFRE model provides valuable general insights, improvements in the spatial resolution of the model to focus on this particular feature would improve the precision of the estimates. Greater temporal detail would also be beneficial but this is difficult when the fishery is only operating for a short time. However, if it does reopen in some of the seamounts that are not protected, then the collection of monitoring data that can be used by the model to provide timely information on ecosystem effects should be considered.

Within the JFRE there is a long-term traditional coastal artisanal tightly-knit fishing community that mainly targets spiny lobsters, J.F. Morwong and recently golden crab. This artisanal fishing has a low impact on the ecosystem and is mainly concentrated in the crustaceans fishery. The lobster fishery represents the greatest artisanal fishing effort and has the greatest impact on the ecosystem. This fishery has generated a significant depletion of the large sized lobster stock with a substantial reduction in the total population biomass. Given the current state of the population, it is not advisable to increase fishing effort. An increase would have a negative effect on catches and financial returns from the fishery. In addition, this increase in fishing effort (irrespective of what it does to catches) may pose a significant problem for the traditional fisheries management of this fishery, breaking down socially enforced management structures. In terms of the ecosystem, the model indicates that a decrease in the abundance of large lobsters has generated an increase in the population of sea urchins. Although this increase does not yet seem to be sizeable, it is still advisable to be careful not to lead the ecosystem towards a regime shift. In addition, it was found that a moderate increase in the fishing effort focused on other species of finfish and crustaceans will not have a significant impact on the ecosystem. Moreover, this change could have a positive socio-economic impact as a result of productive diversification.

This ecosystem model can be used strategically for the management of the JFRE fisheries. Since this is the first ecosystem modeling approach for JFRE, it is advisable to perform some updates especially aimed at reducing uncertainty associated with the input information. Updating information on the trophic relationships among the key species that make up the ecosystem is most urgent. In addition, it is necessary to have time series (monitoring) of the abundance of these key species. While there are some aspects that can be improved, added or updated, this model provides a new and more comprehensive approach for analyzing the current and future status of the JFRE. The JFRE Atlantis model is a comprehensive tool that can provide insights, for example, about the sustainability of the ecosystem under different levels of fishing pressure under a climate change scenario, or to establish which are the main drivers of the ecosystem productivity. In other words, this model is a tool that can help the fisheries managers responsible of this vulnerable marine ecosystem understand its dynamics and its core interactions.

## Supporting information

S1 Supplementary Information Text This file contains the text associated with the supplementary information. (PDF)

S1 Table Functional groups for the JFRE Atlantis model. Each functional group has its identifier (**Code**). In addition, examples of the species that compose these groups are included, with their common and scientific name. The **Management** column describes whether the functional group has any interest for conservation management (**Conservation**) or fisheries management (**Fishery**).
(PDF)

S2 Table Bathymetric range distribution of the JFRE AgeClass functional groups of the Juan Fernández Ridge ecosystem. (PDF)

S3 Table Biomass distribution of orange roughy based on the hydroaccoustic survey [106]. (PDF)

S4 Table Biomass distribution of alfonsino based on the hydroaccoustic survey [106]. (PDF)

S5 Table Distribution of sharks based on the presence of these species in different areas. (PDF)

S6 Table Information used for the predator-prey relationships for the JFRE. The functional group codes correspond to the codes used to identify the functional groups these codes come from S1 Table.
(PDF)

S7 Table Date of release and pelagic larval duration for each modeled functional group. (PDF)

S1 Fig Vertical depth distribution. The ontogenetic stages division for JF. morwong (BRC adult and Juvenil) was based on Rivara 2013 [104].
(PNG)

S2 Fig JF. Morwong horizontal distribution by season. The ontogenetic stages—adults (Left column) and juveniles (right column)—were based on Rivara 2013 [104].
(PNG)

S3 Fig Spiny lobster horizontal distribution by season. The ontogenetic stages—adults (Left column) and juveniles (right column)—were based the size at first maturity estimated by Ernst *et al*. 2016 [50].
(PNG)

S4 Fig Golden crab horizontal distribution by season. The ontogenetic stages—adults (Left column) and juveniles (right column)—were based on size at first maturity estimated by Guerrero and Arana 2009 [105].
(PNG)

S5 Fig Horizontal distribution in Summer for most of the functional groups. The functional group codes are as of S1 Table.
(PNG)

S6 Fig Horizontal distribution in Autumn for most of the functional groups. The functional group codes are as of S1 Table.
(PNG)

S7 Fig Horizontal distribution in Winter for most of the functional groups. The functional group codes are as of S1 Table.
(PNG)

S8 Fig Horizontal distribution in Spring for most of the functional groups. The functional group codes are as of S1 Table.
(PNG)

S9 Fig Time series of the estimated recruitment deviations used for the spiny lobster recruitment model. (PNG)

S10 Fig Time series of rainfall used for forcing the nutrients in Atlantis JFRE. The 90^th^ percentile represents the extreme rainfall events.
(PNG)

S11 Fig Level of connectivity grouped by year and by species for all JFRE. (PNG)

S12 Fig Time series of the biomass relative to the initial biomass for unfished (yellow) and fished (brown) ecosystems. (PNG)

S13 Fig Time series of the proporcion of sea urching in the realized diet of moray eels. (PNG)

S14 Fig Relative effect of moray eels predation on the population of sea urchin. The black dots represent the relative biomass of sea urchin under the predation effect of moray eels. The dotted grey line represents the relative biomass of sea urchin without the predator effect of moray eels. Both time series are relative to the initial biomass of sea urchin.
(PNG)
