# Supplementary material for: The impact of fishing on a highly vulnerable ecosystem, the case of Juan Fernández Ridge ecosystem
Source: PLoS One. 2019 Feb 22;14(2):e0212485. doi: 10.1371/journal.pone.0212485 (PMC6386342; doi:10.1371/journal.pone.0212485)
Supplement: S1 Supplementary Information Text — (PDF) [file pone.0212485.s001.pdf]

Supporting Information : The impact of fishing on a highly vulnerable ecosystem, the case of Juan Fernández Ridge Ecosystem.

Javier Porobic, Elizabeth A. Fulton, Carolina Parada, Stewart Frusher, Billy Ernst, Pablo Manríquez.

*javier.porobicgarate@csiro.au

Supporting information

Initial Conditions

Functional groups

The functional groups are aggregated groups of species with similar size, diet, predators, habitat preferences, migratory patterns and life history strategy [1]. The selection of these ecological groups is mainly determined by the need to capture key ecosystem functional characteristics and management issues (commercial and conservation). In the JFRE Atlantis model there was a *priori* two divisions - either species were of management and conservation concern or they were not (S1 Table). The management functional groups are those that are (were) under some degree of fisheries management or present a conservation concern. The other category of functional groups is composed of groups that have no fisheries component, but are key parts of JFRE.

S1 Table. Functional groups for the JFRE Atlantis model. Each functional group has its identifier (**Code**). In addition, examples of the species that compose these groups are included, with their common and scientific name. The **Management** column describes whether the functional group has any interest for conservation management (**Conservation**) or fisheries management (**Fishery**).

Spatial distribution of functional groups.

Bathymetric range. To establish the range of bathymetric distributions of the individual AgeClass functional groups, a database of fishing and biological monitoring was used; this monitoring has been carried out since 2005 to 2018 on the islands of the Juan Fernández archipelago [2] (JFA; S2 Table). In addition, technical reports and scientific publications were used, supplemented by online information (e.g. Fishbase [3]). For the functional groups for which data were not available, bibliographic information and online database were used.

S2 Table. Bathymetric range distribution of the JFRE AgeClass functional groups of the Juan Fernández Ridge Ecosystem.

Vertical distribution. The vertical distribution by depth layers (8 in the case of JFRE Atlantis) was based on information from the database [2] and reinforced with bibliographic information. For species like the JF. morwong different bathymetric distributions were used for adults and for juveniles (S1 Fig1). This division was based on the size of the first maturity estimated for this species [4]. For benthic species, no analysis was needed, and the vertical distribution was based on the maximum reported depth for that functional group (S2 Table).

S1 Fig. Vertical depth distribution. The ontogenetic stages division for JF. morwong (BRC adult and Juvenil) was based on Rivara 2013 [4]

Horizontal distribution. To establish the horizontal and seasonal distribution of the functional groups (by polygons) we used the information coming from the fisheries and biological databases available for the JFA [2, 5] (S1-S8 Figs). In addition, we used information contained in technical reports and scientific publications was utilised (S3-S5 Tables). For species that do not have an abundance index per zone, only information

reflecting the presence or absence of these species was used (S4 Table). Therefore, for these species the total initial biomass obtained was evenly distributed between the different areas.

S2 Fig. JF. Morwong horizontal distribution by season. The ontogenetic stages - adults (Left column) and juveniles (right column) - were based on Rivara 2013 [4]

S3 Fig. Spiny lobster horizontal distribution by season. The ontogenetic stages - adults (Left column) and juveniles (right column) - were based the size at first maturity estimated by Ernst *et al.* 2016 [2]

S4 Fig. Golden crab horizontal distribution by season. The ontogenetic stages - adults (Left column) and juveniles (right column) - were based on size at first maturity estimated by Guerrero and Arana 2009 [6]

S5 Fig. Horizontal distribution in Summer for most of the functional groups. The functional group codes are as of S1 Table.

S6 Fig. Horizontal distribution in Autumn for most of the functional groups. The functional group codes are as of S1 Table.

S7 Fig. Horizontal distribution in Winter for most of the functional groups. The functional group codes are as of S1 Table.

S8 Fig. Horizontal distribution in Spring for most of the functional groups. The functional group codes are as of S1 Table.

S3 Table. Biomass distribution of orange roughy based on the hydroaccoustic survey [7].

S4 Table. Biomass distribution of alfonsino based on the hydroaccoustic survey [7]

S5 Table. Distribution of sharks based on the presence of these species in different areas

Diet in the Juan Fernández Ridge Ecosystem

Unfortunately for the JFRE, information on the trophic relationships and diet form most functional groups is scarce or nonexistent. Due to this, to configure the model, it was necessary to carry out a bibliographic search to reconstruct the diets of the species that make up the JFRE. For the groups where it was impossible to find any information at the species level, complementary information of congeneric species was used (S6 Table).

S6 Table. Information used for the predator-prey relationships for the JFRE. The functional group codes correspond to the codes used to identify the functional groups these codes come from S1 Table.

Forcings

Recruitment deviations

The time series of the deviations used to force the lobster recruitment (S9 Fig) came from a population dynamics model of spiny lobster [8]. This model is based on the size structured model presented by Sullivan et al. [9] and divides the demographic processes in to three parts: i) recruitment, modeled through a stock-recruit relationship with a random component (recruitment deviations); ii) natural and fishing mortality; and iii) somatic growth, modeled by a growth transition matrix. The calculation of the recruitment during year t is defined as the number of individuals generated by the spawning stock that recruit to the population, this relationship is defined by:

$$R_t = \frac{S_t}{(\delta + \lambda S_t)} e^{\epsilon_t} \tag{1}$$

In this equation S_t corresponds to the spawning stock at the end of year t, ϵ_t corresponds to the associated error (recruitment deviations), δ and $lambda$ are the parameters of the stock recruit function, which under a reparametrization are defined by:

$$\delta = \frac{S_0}{R_0}(1 - \frac{z - 0.2}{0.8z}) \tag{2}$$

$$\lambda = \frac{z - 0.2}{0.8 z R_0} \quad (3)$$

Where R_0 corresponds to the virginal Recruitment, S_0 to the virgin spawning stock and z corresponds to the dependent parameter of the stock-recruit function which can vary between values of 0.2 and 1.

S9 Fig. Time series of the estimated recruitment deviations used for the spiny lobster recruitment model.

Rainfall forcing

The time series of nutrient contribution to the ecosystem (mainly NO3) was based on rainfall time series reconstruction around the JFRE islands [10] (S10 Fig). The reconstruction of the gaps in time series was done using an ARIMA-Kalman smoothing and state space models [11]. To represent the events of major injection of nutrients to JFRE due to flooding, especially around the islands, only the extreme rain events were used. Only those events located above the 90th percentile were considered extreme events (S10 Fig).

S10 Fig. Time series of rainfall used for forcing the nutrients in Atlantis JFRE. The 90th percentile represents the extreme rainfall events.

Connectivity Model

Areas and date of release and settlement A particle tracking method was used to determine the connectivity matrix used to force larval dispersal in the model (S11 Fig). The areas of particle release were based on the observed spatial distribution of the species (S1 *Spatial distribution of functional groups*). 1200 particles were released for each release zone, corresponding to the minimum number of particles released to achieve stable results. The release dates and the larval duration were established based on the average spawning dates and the average duration times for each functional group - see S7 Table. Settlement areas were defined as the places where adults live and areas that are close to other seamounts or islands. This procedure was undertaken so that Atlantis could be used to explore other places where the functional groups could live. This was necessary due to the scarcity of spatial distribution information of the functional groups.

S11 Fig. Level of connectivity Grouped by year and by species for all

JFRE.

S7 Table. Date of release and pelagic larval duration for each modeled functional group

Additional results

Relative Biomass

The time series in (S12 Figure) represents the trajectories of relative biomass for all the functional groups. There are marked seasonal variations, which are mainly related to processes of population recruitment. There are also species displaying a marked trend in the population biomass (e.g. sea urchin and mollusc), which are related to the forcing of lobster dynamics (recruitment deviations). In general, there is a strong similarity between the model trajectories with and without fishing, with the exception of the small number of species that are most affected by fishing activity. The target species present the most marked variations (i.e. spiny lobster, J.F. morwong, alfonsino and orange roughy). In addition, some of the non-target species are also affected indirectly by bycatch, which leads to small variations in their biomasses.

S12 Fig. Time series of the biomass relative to the initial biomass for unfished (yellow) and fished (brown) ecosystems.

Moray eels realized diet

The time series in (S13 Figure) represents the increase through the time of sea urchin as a prey in the realized diet of moray eels. This time series shows a high inverse correlation with spiny lobster's biomass (spearman.cor = -0.682, p-value $< 2.2e-12$). This suggests that changes in the biomass of spiny lobster have an effect in the effective consumption of sea urchin by moray eels. This indicates that part of the biomass of sea urchin that is not being consumed by spiny lobster is been transferred to the diet of Moray eels.

S13 Fig. Time series of the proporcion of sea urching in the realized diet of moray eels.

Effect of moray eels on sea urchin population

The time series in (S14 Fig) shows the effect of moray eels on the population of sea urchin. It is clearly observed that when there is a direct trophic relation between moray eels (predator) and the sea urchin (prey) population. In a scenario without the predation effect of moray eels on sea urchins, the urchins biomass grows on average of 18% to 21%. This result reinforces the thesis that the moray eels may play an important role in controlling the sea urchin population.

S14 Fig. Relative effect of moray eels predation on the population of sea urchin. The black dots represent the relative biomass of sea urchin under the predation effect of moray eels. The dotted grey line represents the relative biomass of sea urchin without the predator effect of moray eels. Both time series are relative to the initial biomass of sea urchin.

References

1. Fulton E, Smith A, Smith D. Alternative Management Strategies for Southeast Australian Commonwealth Fisheries: Stage 2: Quantitative Management Strategy Evaluation. CSIRO; 2007. June.

2. Ernst B, Rivara P, Tapia B, Santa Cruz F, Espinoza L, Manríquez P, et al. Evaluación directa de la Breca (Nemadactylus gayi, Kner 1865) en torno a las islas Robinson Crusoe y Santa Clara. Departamento de Oceanografía - Universidad de Concepción; 2016.

3. Froese R, Pauly D. Fishbase; 2017. Available from: `www.fishbase.org`.

4. Rivara P. Estudio de la biología reproductiva de Nemadactylus gayi (Kner 1865), en el Archipiélago Juan Fernandez. University of Concepción; 2013.

5. Friedlander AM, Ballesteros E, Caselle JE, Gaymer CF, Palma AT, Petit I, et al. Marine biodiversity in Juan Fernández and Desventuradas islands, Chile: Global endemism hotspots. PLoS ONE. 2016;11(1). doi:10.1371/journal.pone.0145059.

6. Guerrero A, Arana P. Size structure and sexual maturity of the golden crab (Chaceon chilensis) exploited off Robinson Crusoe Island, Chile. Latin American

Journal of Aquatic Research. 2009;37(3):347–360. doi:10.3856/vol37-issue3-fulltext-6.

7. Niklitschek E, Cornejo J, Hernández E, Toledo P, Herranz C, Merino R, et al. Informe Final: Evaluación hidroacustica del alfonsino y orange roughy, año 2006. Universidad Austral; 2007.

8. Porobic J, Canales-Aguirre CB, Ernst B, Galleguillos R, Hernández CE. Biogeography and historical demography of the juan fernández rock lobster, jasus frontalis (Milne Edwards, 1837). Journal of Heredity. 2013;104(2):223–233. doi:10.1093/jhered/ess141.

9. Sullivan PJ, Lai HL, Gallucci VF. A Catch-at-Length Analysis that Incorporates a Stochastic Model of Growth. Canadian Journal of Fisheries and Aquatic Sciences. 1990;47(1):184–198. doi:10.1139/f90-021.

10. Directorate General of Civil Aeronautics of Chile. Climatological yearbooks;. Available from: `http://164.77.222.61/climatologia/php/menuAnuarios.php`.

11. Shumway RH, Stoffer DS. Time Series Analysis and Its Applications With R Examples; 2011.
