# Supplementary material for: The impact of fishing on a highly vulnerable ecosystem, the case of Juan Fernández Ridge ecosystem
Source: PLoS One. 2019 Feb 22;14(2):e0212485. doi: 10.1371/journal.pone.0212485 (PMC6386342; doi:10.1371/journal.pone.0212485)
Supplement: S1 Table — Each functional group has its identifier (Code). In addition, examples of the species that compose these groups are included, with their common and scientific name. The Management column describes whether the functional group has any interest for conservation management (Conservation) or fisheries management (Fishery). (PDF) [file pone.0212485.s002.pdf]

S1 Table 1. Functional groups for the JFRE Atlantis model. Each functional group has its identifier (**Code**). In addition, examples of the species that compose these groups are included, with their common and scientific name. The **Management** column describes whether the functional group has any interest for conservation management (**Conservation**) or fisheries management (**Fishery**).

Code	Functional groups	Common name	Scientific Name	Management
**SPL**	Spiny lobster	Langosta de J.F.	*Jasus frontalis*	Fishery
**GCR**	Golden crab	Cangrejo Dorado	*Chaceon chilensis*	Fishery
**BRC**	J.F. morwong	Breca	*Nemadactylus gayi*	Fishery
**ANG**	Morey eels	Anguila	*Gymnothorax porphyreus*	Fishery
**VID**	Yellowtail amberjack	Vidriola	*Seriola lalandi*	Fishery
**ALF**	Alfonsino	Alfonsino	*Beryx splendens*	Fishery
**ORO**	Orange roughy	Orange Roughy	*Hoplostethus atlanticus*	Fishery
**OCT**	Octopus	Pulpo de J.F.	*Octopus crusoe*	Fishery
**CHO**	Sharks	Tollo	*Squalus mitsukuri*	-
**OTA**	Fur seals	Lobo de J.F.	*Actocephalus phillippi*	Conservation
**SPF**	Small pelagic fish	Pampanito	*Scorpis chilensis*	Fishery
		Graniento	*Caprodon longimanus*	
**LPF**	Large pelagic fish	Corvina de J.F.	*Umbrina reedi*	Fishery
		Jurel de J.F.	*Pseudocaranx chilensis*	
		Sierra	*Thyrsites atun*	
**SBF**	Small benthic fish	Colorado	*Plectranthias exsul*	Fishery
		Cabrilla	*Chironemus delfini*	
		Chancharro	*Helicolenus lengerichi*	
		Pez Mariposa	*Pterygotrigla picta*	
		Jerguilla de J.F.	*Girella albostriata*	
**LBF**	Large benthic fish	Bacalado de J.F.	*Polyprion oxigeneios fernandezianus*	Fishery
		Lenguado	*Paralichthys fernandezianus*	
		Congrio	*Lotella fernandeziana*	
**MOL**	Mollusca	Caracol blanco	*Fusitritron magellanicum*	-
		Ostra	*Nucula fernandeziana*	
		Loco	*Concholepas sp*	
**BFF**	Deposit feeders	Poliquetos	*Scoloplos juanfernandezensis*	-
**SCR**	Other crustacean	Centolla de J.F.	*Paraloma rathbuni*	-
		Jaiva	*Talifrus dentatus*	
**SUR**	Sea urchin	Erizo	*Centrostephanus rodgersii*	-
		Estrella de mar	*Patiriella calcarata*	
**COR**	Coral	Coral Piedra	*Desmophyllum dianthus*	Conservation
		Coral Negro	*Bathyphates patula*	
**SZO**	Small zooplankton	microzooplankton	Several species	-
**MZO**	Medium zooplankton	Copepods	*Canthocalanus pauper*	-
**LZO**	Large zooplankton	Euphausiid	*Euphausia mucronata*	-
**MA**	Macroalgae	Alga verde	*Ulva intestinalis*	-
**SPH**	Small phytoplankton	Nano-microplankton	Several species	-
**LPH**	Large phytoplankton	Diatom	Several species	-
