# Supplementary material for: The impact of fishing on a highly vulnerable ecosystem, the case of Juan Fernández Ridge ecosystem
Source: PLoS One. 2019 Feb 22;14(2):e0212485. doi: 10.1371/journal.pone.0212485 (PMC6386342; doi:10.1371/journal.pone.0212485)
Supplement: S2 Table — (PDF) [file pone.0212485.s003.pdf]

S1 Table 2. Bathymetric range distribution of the JFRE AgeClass functional groups of the Juan Fernández Ridge Ecosystem.

Functional Groups	Minimum Depth	Maximum Depth	Reference
SPL	2	250	[1, 2]
GCR	100	1000	[1, 3]
BRC	20	300	[1, 4]
ORO	500	1000	[5]
ALF	200	600	[5]
LPF	0	200	[1]
SPF	0	250	[1]
SBF	0	250	[1]
LBF	0	250	[1]
VID	3	150	[1, 6]
ANG	0	300	[1, 3]
CHO	20	600	[6, 6, 6, 7]
DOL	0	200	[6]
COR	50	1000	[1, 8–10]
MPF	0	5000	[6, 9]

References

1. Ernst B, Rivara P, Tapia B, Santa Cruz F, Espinoza L, Manríquez P, et al. Evaluación directa de la Breca (Nemadactylus gayi, Kner 1865) en torno a las islas Robinson Crusoe y Santa Clara. Departamento de Oceanografía - Universidad de Concepción; 2016.

2. Retamal MA, Arana PM. Descripción y distribución de cinco crustáceos decápodos recolectados en aguas profundas en torno a las islas Robinson Crusoe y Santa Clara (Archipiélago de Juan Fernández, Chile). Investigaciones marinas. 2000;28:149–163. doi:10.4067/S0717-71782000002800011.

3. Arana PM. Pesca exploratoria con trampas alrededor de las islas Robinson Crusoe y Santa Clara, archipiélago de Juan Fernández, Chile. Investigaciones marinas. 2000;28:39–52. doi:10.4067/S0717-71782000002800005.

4. Arana PM, Vega R. Pesca exploratoria con espineles en aguas profundas en torno a la isla Robinson Crusoe (Archipiélago de Juan Fernández), Chile. Investigaciones marinas. 2000;28:219–230. doi:10.4067/S0717-71782000002800016.

5. Niklitschek E, Cornejo J, Hernández E, Toledo P, Herranz C, Merino R, et al. Informe Final: Evaluación hidroacustica del alfonsino y orange roughy, año 2006. Universidad Austral; 2007.

6. Froese R, Pauly D. Fishbase; 2017. Available from: `www.fishbase.org`.

7. Andrade I, Pequeño G. Mesobathic chondrichthyes of the Juan Fernández seamounts: Are they different from those of the central Chilean continental slope? Revista de Biologia Tropical. 2008;56(1):181–190.

8. Niklitschek E, Cornejo-Donoso J, Oyarzún C, Hernández E, Toledo P. Developing seamount fishery produces localized reductions in abundance and changes in species composition of bycatch. Marine Ecology. 2010;31(SUPPL. 1):168–182. doi:10.1111/j.1439-0485.2010.00372.x.

9. Yañez E, Silva C, Vega R, Alvarez L, Silva N, Palma S, et al. Biodiversidad De Montes Submarinos. Universidad de Vaparaiso; 2008. Available from: `http://www.fip.cl/FIP/Archivos/pdf/informes/inffinal2006-57.pdf`.

10. Friedlander AM, Ballesteros E, Caselle JE, Gaymer CF, Palma AT, Petit I, et al. Marine biodiversity in Juan Fernández and Desventuradas islands, Chile: Global endemism hotspots. PLoS ONE. 2016;11(1). doi:10.1371/journal.pone.0145059.
