# Supplementary material for: The impact of fishing on a highly vulnerable ecosystem, the case of Juan Fernández Ridge ecosystem
Source: PLoS One. 2019 Feb 22;14(2):e0212485. doi: 10.1371/journal.pone.0212485 (PMC6386342; doi:10.1371/journal.pone.0212485)
Supplement: S3 Table — (PDF) [file pone.0212485.s004.pdf]

S1 Table 3. Biomass distribution of orange roughy based on the hydroaccoustic survey [?].

Geographical area	2003	2004	2005	2006	Distribution (%)	JFRE Atlantis polygons
Seamount - JF1	2196	2019	9590	3749	29%	31,32,33,34
Seamount - JF2	7246	6062	2847	2201	31%	30
Seamount - JF3	4536	1905	1531	1565	16%	29
Seamount - JF4	2981	1572	1586	492	11%	28
Seamount - BO1		927	1813		9%	50
Seamount - BO2		654	536		4%	49
