# Supplementary material for: The impact of fishing on a highly vulnerable ecosystem, the case of Juan Fernández Ridge ecosystem
Source: PLoS One. 2019 Feb 22;14(2):e0212485. doi: 10.1371/journal.pone.0212485 (PMC6386342; doi:10.1371/journal.pone.0212485)
Supplement: S4 Table — (PDF) [file pone.0212485.s005.pdf]

S1 Table 4. Biomass distribution of alfonsino based on the hydroaccoustic survey [?]

Geographical area	2005	2006	Distribution (%)	JFRE Atlantis polygons
Seamount - JF1	7608	11258	47.5%	31,32,33,34
Seamount - JF2	10646	3112	34.6%	30
Seamount - JF4	181	203	1.0%	28
Seamount - JF6		3352	16.9%	47
