# Supplementary material for: The impact of fishing on a highly vulnerable ecosystem, the case of Juan Fernández Ridge ecosystem
Source: PLoS One. 2019 Feb 22;14(2):e0212485. doi: 10.1371/journal.pone.0212485 (PMC6386342; doi:10.1371/journal.pone.0212485)
Supplement: S5 Table — (PDF) [file pone.0212485.s006.pdf]

S1 Table 5. Distribution of sharks based on the presence of these species in different areas

Zone	Zones	Reference
Seamount - JF1	31,32,33,34	[1]
Seamount - JF2	30	[1]
Seamount - JF3	29	[2]
Seamount - JF4	28	[2]
Robinson Is.	35,36,37,38	[3]
Selkirk Is.	41,42,43,44	[3]

References

1. Yañez E, Silva C, Vega R, Alvarez L, Silva N, Palma S, et al. Biodiversidad De Montes Submarinos. Universidad de Vaparaiso; 2008. Available from: http://www.fip.cl/FIP/Archivos/pdf/informes/inffinal2006-57.pdf.

2. Andrade I, Pequeño G. Mesobathic chondrichthyes of the Juan Fernández seamounts: Are they different from those of the central Chilean continental slope? Revista de Biologia Tropical. 2008;56(1):181–190.

3. Arana PM. Experiencias de pesca con red de enmalle en las islas Robinson Crusoe y Santa Clara, Chile. Investigaciones marinas. 2000;28:231–237. doi:10.4067/S0717-71782000002800017.
