# Supplementary material for: The impact of fishing on a highly vulnerable ecosystem, the case of Juan Fernández Ridge ecosystem
Source: PLoS One. 2019 Feb 22;14(2):e0212485. doi: 10.1371/journal.pone.0212485 (PMC6386342; doi:10.1371/journal.pone.0212485)
Supplement: S6 Table — The functional group codes correspond to the codes used to identify the functional groups these codes come from S1 Table. (PDF) [file pone.0212485.s007.pdf]

S2 Table 7. Information used for the predator-prey relationships for the JFRE. The functional group codes correspond to the codes used to identify the functional groups these codes come from S1 Table 1.

Function Group Code	Species	Reference
SPL	*Jasus lalandii*	[1, 2]
	Jasus edwearsii	[3–5]
GCR	*Chaceon notialis*	[6]
	Chaceon quinquedens	[6]
	Chaceon ramosae	[6]
BRC	*Nemadactilys gayi*	[7, 8]
VID	*Seriola lalandi*	[9, 10]
ORO	*Hoplostethus atlanticus*	[6, 11–14]
ALF	*Beryx splendens*	[6, 15, 16]
ANG	*Lycodontis porphyreus*	[17]
	Gymnothorax vicinus	[18]
CHO	*Deep sea Squalus*	[14]
	Squalus fernandinus	[19]
	Squalus mitsukurii	[6, 20]
OTA	*Arctocephalus philippii*	[21–23]
DOL	*Delphinus delphis*	[6, 24, 25]
	Tursiops truncatus	[6]
	Globicephala macrorhynchus	[6]
BIR	*Puffinus cretopus*	[26]
SQD	*Dosidicus gigas*	[6]
	vampyroteuthis infernalis	[6]
OCT	*Octopus crusoe*	[27]
	Octopus mimus	[6]
	Enteroctopus megalocyathus	[28]
	Octopus vulgaris	[29]
LPF	*Psudocaranx chilensis*	[7]
	Psudocaranx chilensis	[6]

S2 Table 7. continuation.

Function Group Code	Species	Reference
SPF	*Malapterus reticulatus*	[27]
	Scorpix chilensis	[7]
	Malapterus reticulatus	[7]
	Thyrsites atun	[6]
	Callanthias sp.	[6]
SBF	*Hipoplectrodes semicinctum*	[7]
	Plectranthias sp	[6]
	Aseraggodes sp.	[6]
	Girella albostriata	[6]
MPF	Myctophidae	[6]
LBF	*Polyprion oxygeneios*	[17]
	Aserragodes macleayanus	[30]
	Polyprion oxygeneios	[6]
	Paralabrax sp.	[6]
	Paralichthys sp.	[6]
SZO	Microzooplankton	[31, 32]
MZO	Mesozooplankton	[33–36]
LZO	Euphausiid	[37]
SCR	*Projasus bahamondei*	[38]
	Paromola cuviieri	[6]
	Chaceon quinquedens	[39]
	Ovalipes trimaculatus	[6]
BFF	Polychaete	[40]
MOL	*Acanthopleura*	[41]
	Concholepas concholepas	[42]
	Arca sp.	[6]
	Aplysia sp.	[6]
SUR	Sea Urchin	[6]
	Actinidae	[6]
COR	Deep sea coral	[43]

References

1. Van Zyl RF, Mayfield S, Pulfrich A, Griffiths CL. Predation by West Coast rock lobsters (Jasus lalandii) on two species of winkle (Oxystele sinemis and Turbo cidaris). South African Journal of Zoology. 1998;33(4):203–209. doi:10.1080/02541858.1998.11448473.

2. Haley CN, Blamey LK, Atkinson LJ, Branch GM. Dietary change of the rock lobster Jasus lalandii after an 'invasive' geographic shift: Effects of size, density and food availability. Estuarine, Coastal and Shelf Science. 2011;93(2):160–170. doi:10.1016/j.ecss.2011.04.015.

3. Guest MA, Frusher SD, Nichols PD, Johnson CR, Wheatley KE. Trophic effects of fishing southern rock lobster Jasus edwardsii shown by combined fatty acid and stable isotope analyses. Marine Ecology Progress Series. 2009;388:169–184. doi:10.3354/meps08096.

4. Connell SC, O'Rorke R, Jeffs AG, Lavery SD. DNA identification of the phyllosoma diet of Jasus edwardsii and Scyllarus sp. Z. New Zealand Journal of Marine and Freshwater Research. 2014;48(3):416–429. doi:10.1080/00288330.2014.914042.

5. O'Rorke R, Lavery SD, Wang M, Nodder SD, Jeffs AG. Determining the diet of larvae of the red rock lobster (Jasus edwardsii) using high-throughput DNA sequencing techniques. Marine Biology. 2014;161(3):551–563. doi:10.1007/s00227-013-2357-7.

6. Froese R, Pauly D. Fishbase; 2017. Available from: `www.fishbase.org`.

7. Ramírez F. Estructura trófica, abundancia y ecomorfología del ensamble de peces costeros de la isla Robinson Crusoe, archipiélago de Juan Fernández, Chile Insular. Universidad Andres Bello; 2012.

8. Ernst B, Oyarzún C, Vilches J, Rivara P, Tapia B, Alvarez C, et al. Final Report: Estudio Ecosistémico de las pesquerías del sitio piloto Archipiélago de Juan Fernández. Fase Metodológica. Universidad de Concepción; 2015.

9. Vergani M, Acha EM, de Astarloa JMD, Giberto D. Food of the yellowtail amberjack Seriola lalandi from the south-west Atlantic. Journal of the Marine Biological Association of the UK. 2008;88(04):851–852. doi:10.1017/S0025315408000477.

10. Bray D. Online Catalog: Fishes of Australia- Yellowtail Kingfish.; 2018. Available from: `http://fishesofaustralia.net.au/home/species/1662/{#}summary`.

11. Bulman CM, Koslow JA. Diet and food consumption of a deep-sea fish, orange roughy Hoplostethus atlanticus (Pisces: Trachichthyidae), off southeastern Australia. Marine Ecology Progress Series. 1992;82:115–129. doi:10.3354/meps082115.

12. Labbé JF, Arana P. Alimentación de orange roughy, Hoplostethus atlanticus (Pisces: Trachichthyidae), en el archipiélago de Juan Fernández, Chile. Revista de Biologia Marina y Oceanografia. 2001;36(1):75–82. doi:10.4067/S0718-19572001000100007.

13. Niklitschek E, Canales-Aguirre C, Ferrada S, Galleguillos R, George-nascimento M, Lafon A, et al. Unidades poblacionales de orange roughy (Hoplostethus atlanticus). Coyaique: Universidad Austral de Chile; 2009.

14. Dunn MR, Forman JS. Hypotheses of spatial stock structure in orange roughy Hoplostethus atlanticus inferred from diet, feeding, condition, and reproductive activity. PLoS ONE. 2011;6(11). doi:10.1371/journal.pone.0026704.

15. Dürr J, González JA. Feeding habits of Beryx splendens and Beryx decadactylus (Berycidae) off the Canary Islands. Fisheries Research. 2002;54(3):363–374. doi:10.1016/S0165-7836(01)00269-7.

16. Horn PL, Forman J, Dunn MR. Feeding habits of alfonsino Beryx splendens. Journal of Fish Biology. 2010;76(10):2382–2400. doi:10.1111/j.1095-8649.2010.02630.x.

17. Pizarro MF, Tiffou M. La langosta de Juan Fernández III. Sinopsis sobre la biología de la langosta de Juan Fernández Jasus frontalis (Milne-Edwards, 1837). Investigaciones Marinas Valparaíso. 1974;5(1):1–52.

18. Young RF, Winn HE. Activity Patterns , Diet , and Shelter Site Use for Two Species of Moray Eels , Gymnothorax moringa and Gymnothorax vicinus , in Belize Published by : American Society of Ichthyologists and Herpetologists (ASIH) Stable URL : http://www.jstor.org/stable/. American Society of Ichthyologists and Herpetologists. 2003;2003(1):44–55.

19. Arana P. Juan Fernández Islands (Chile) Living Marine Resources and Fishery Perspective. School of Oceanography - Oregon State University; 1979.

20. Cortes E. Standardized diet compositions and trophic levels of sharks. ICES Journal of Marine Science. 1999;56(May):707–717. doi:10.1006/jmsc.1999.0489.

21. Acuna HO, Francis JM. Spring and summer prey of the Juan Fernandez fur seal, Arctocephalus philippii. Canadian Journal of Zoology. 1995;73(8):1444–1452.

22. Osman L. Population status, distribution, and foraging ecology of Arctocephalus philippi (Peter 1866) At Juan Fernández Archipelago [PhD.]. Universidad Austral de Chile; 2007.

23. Diaz P. Dieta de las hembras de Arctocephalus philippii (Peters, 1866) durante la temporada reproductiva en el Archipielago de Juan Fernandez. [Undergrad]. Universidad Austral; 2007.

24. Pusineri C, Magnin V, Meynier L, Spitz J, Hassani S, Ridoux V. Food and feeding ecology of the common dolphin (Delphinus delphis) in the oceanic Northeast Atlantic and comparison with its diet in neritic areas. Marine Mammal Science. 2007;23(1):30–47. doi:10.1111/j.1748-7692.2006.00088.x.

25. Brophy JT, Murphy S, Rogan E. The diet and feeding ecology of the short-beaked common dolphin (Delphinus delphis) in the northeast Atlantic. Report of the International Whaling Commission. 2006;(1999):1–18.

26. Azocar J, García M, Colodro V, Arata J, Hodum P, Morgan K. Pink-footed Shearwater Puffinus creatopus. Seventh Meeting of the Advisory Committee. La Rochelle: Agreement on the conservation of; 2013. May.

27. Petit IJ, Gaymer CF, Palma ÁT, Wahle RA. Predation of Juvenile <i>Jasus frontalis</i> : An Endemic Spiny Lobster of the Juan Fernández Archipelago,

Chile. Journal of Shellfish Research. 2015;34(3):1085–1089. doi:10.2983/035.034.0335.

28. Ibáñez CM, Chong JV. Feeding ecology of Enteroctopus megalocyathus (Cephalopoda: Octopodidae) in southern Chile. Journal of the Marine Biological Association of the UK. 2008;88(04):793–798. doi:10.1017/S0025315408001227.

29. Smith CD. Diet of Octopus vulgaris in False Bay, South Africa. Marine Biology. 2003;143(6):1127–1133. doi:10.1007/s00227-003-1144-2.

30. Sumpton W, Greenwood J. Pre- and post-flood feeding ecology of four species of juvenile fish from the logan-albert estuarine system, moreton bay, queensland. Marine and Freshwater Research. 1990;41(6):795–806. doi:10.1071/MF9900795.

31. Calbet A. The trophic roles of micro zooplankton in marine systems. ICES Journal of Marine Science. 2008;65(May):325–331.

32. Burian A, Schagerl M, Yasindi A. Microzooplankton feeding behaviour: Grazing on the microbial and the classical food web of African soda lakes. Hydrobiologia. 2013;710(1):61–72. doi:10.1007/s10750-012-1023-2.

33. Batten SD, Fileman ES, Halvorsen E. The contribution of microzooplankton to the diet of mesozooplankton in an upwelling filament off the north west coast of Spain. Progress in Oceanography. 2001;51(2-4):385–398. doi:10.1016/S0079-6611(01)00076-3.

34. Bode A, Alvarez-Ossorio MT. Taxonomic versus trophic structure of mesozooplankton: A seasonal study of species succession and stable carbon and nitrogen isotopes in a coastal upwelling ecosystem. ICES Journal of Marine Science. 2004;61(4):563–571. doi:10.1016/j.icesjms.2004.03.004.

35. Wu CJ, Chiang KP, Liu H. Diel feeding pattern and prey selection of mesozooplankton on microplankton community. Journal of Experimental Marine Biology and Ecology. 2010;390(2):134–142. doi:10.1016/j.jembe.2010.05.003.

36. Motwani NH, Gorokhova E. Mesozooplankton grazing on picocyanobacteria in the baltic sea as inferred from molecular diet analysis. PLoS ONE. 2013;8(11). doi:10.1371/journal.pone.0079230.

37. Antezana T. Euphausia mucronata: A keystone herbivore and prey of the Humboldt Current System. Deep-Sea Research Part II: Topical Studies in Oceanography. 2010;57(7-8):652–662. doi:10.1016/j.dsr2.2009.10.014.

38. Arana P. Chilean jagged lobster, Projasus bahamondei, in the southeastern Pacific Ocean: current state of knowledge. Lat Am J Aquat Res,. 2014;42(1):1–17. doi:103856/vol42-issue1-fulltext-1.

39. Steimle FW, Zetlin Ca, Chang S. ESSENTIAL FISH HABITAT SOURCE DOCUMENT: Red deepsea crab, Chaceon (Geryon) quinquedens, life history and habitat characteristics. NOAAA Technical Memoradum NMFS-NE-163. 2001;163(January):1–36.

40. Jumars PA, Dorgan KM, Lindsay SM. Diet of Worms Emended: An Update of Polychaete Feeding Guilds. Annual Review of Marine Science. 2015;7(1):497–520. doi:10.1146/annurev-marine-010814-020007.

41. Camus PA, Navarrete AH, Sanhueza ÁG, Felipe Opazo L. Trophic ecology of the chiton Acanthopleura echinata on Chilean rocky shores. Revista Chilena de Historia Natural. 2012;85(1):123–135. doi:10.4067/S0716-078X2012000100010.

42. Stotz WB, Gonzalez S, Caillaux L, Aburto J. Quantitative evaluation of the diet and feeding behavior of the carnivorous Gatropod,Concholepas concholepas (Bruguiere,1789) (Muricidae) in subtidal habitats in the southeastern Pacific upwelling system. Journal of Shellfish Research. 2003;22(1):147–164. doi:10.2983/035.029.0302.

43. Kiriakoulakis K, Fisher EL, Wolff GA, Freiwald A, Grehan A, Roberts JM, et al. Lipids and nitrogen isotopes of two deep-water corals from the North-East Atlantic: initial results and implications for their nutrition. Cold-water Corals and Ecosystems. 2005; p. 715–729. doi:10.1007/3-540-27673-$4_3$7.
