# Supplementary material for: The impact of fishing on a highly vulnerable ecosystem, the case of Juan Fernández Ridge ecosystem
Source: PLoS One. 2019 Feb 22;14(2):e0212485. doi: 10.1371/journal.pone.0212485 (PMC6386342; doi:10.1371/journal.pone.0212485)
Supplement: S7 Table — (PDF) [file pone.0212485.s008.pdf]

S3 Table 1. Date of release and pelagic larval duration for each modeled functional group.

Functional group	Starting Date	PLD
ALF	01/January	150
ANG	01/December	450
BRC	01/June	365
GCR	01/October	200
LBF	01/August	365
LPF	01/January	365
ORO	01/August	200
SBF	01/August	365
SPF	01/August	365
SPL	01/October	365
