# Supplementary figures and images for: The impact of fishing on a highly vulnerable ecosystem, the case of Juan Fernández Ridge ecosystem
Source: PLoS One. 2019 Feb 22;14(2):e0212485. doi: 10.1371/journal.pone.0212485 (PMC6386342; doi:10.1371/journal.pone.0212485)

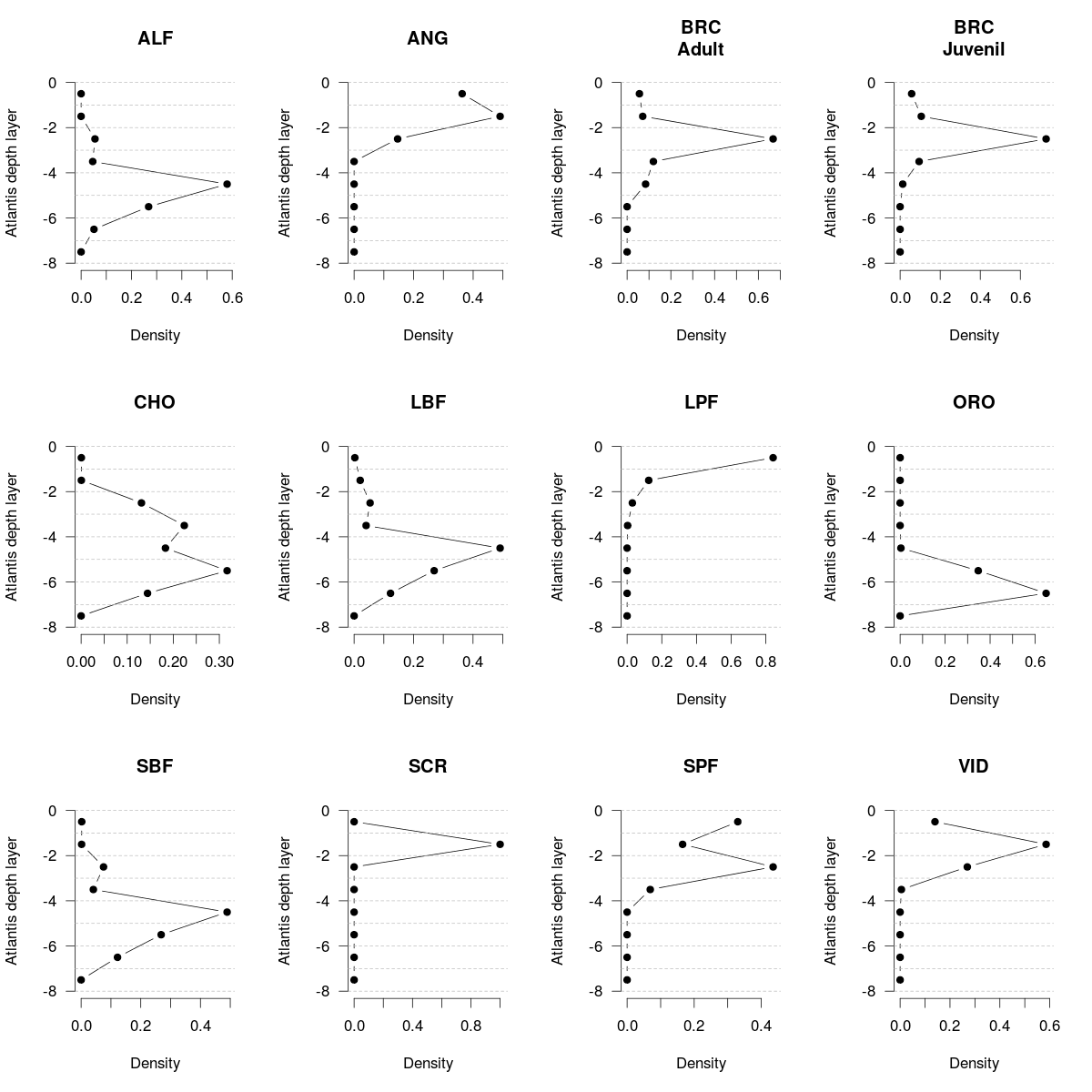

Supplement: S1 Fig — The ontogenetic stages division for JF. morwong (BRC adult and Juvenil) was based on Rivara 2013 [104]. (PNG) [file pone.0212485.s009.png]

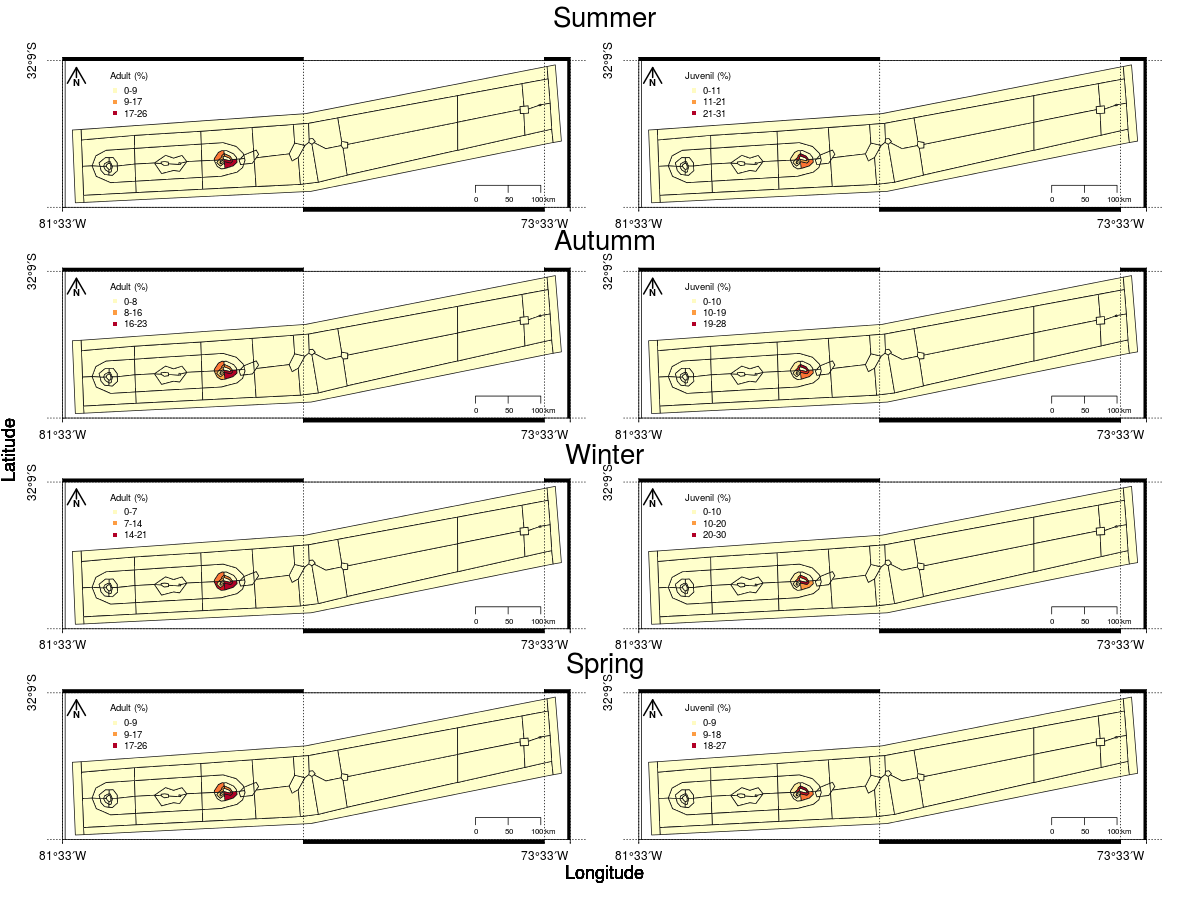

Supplement: S2 Fig — The ontogenetic stages—adults (Left column) and juveniles (right column)—were based on Rivara 2013 [104]. (PNG) [file pone.0212485.s010.png]

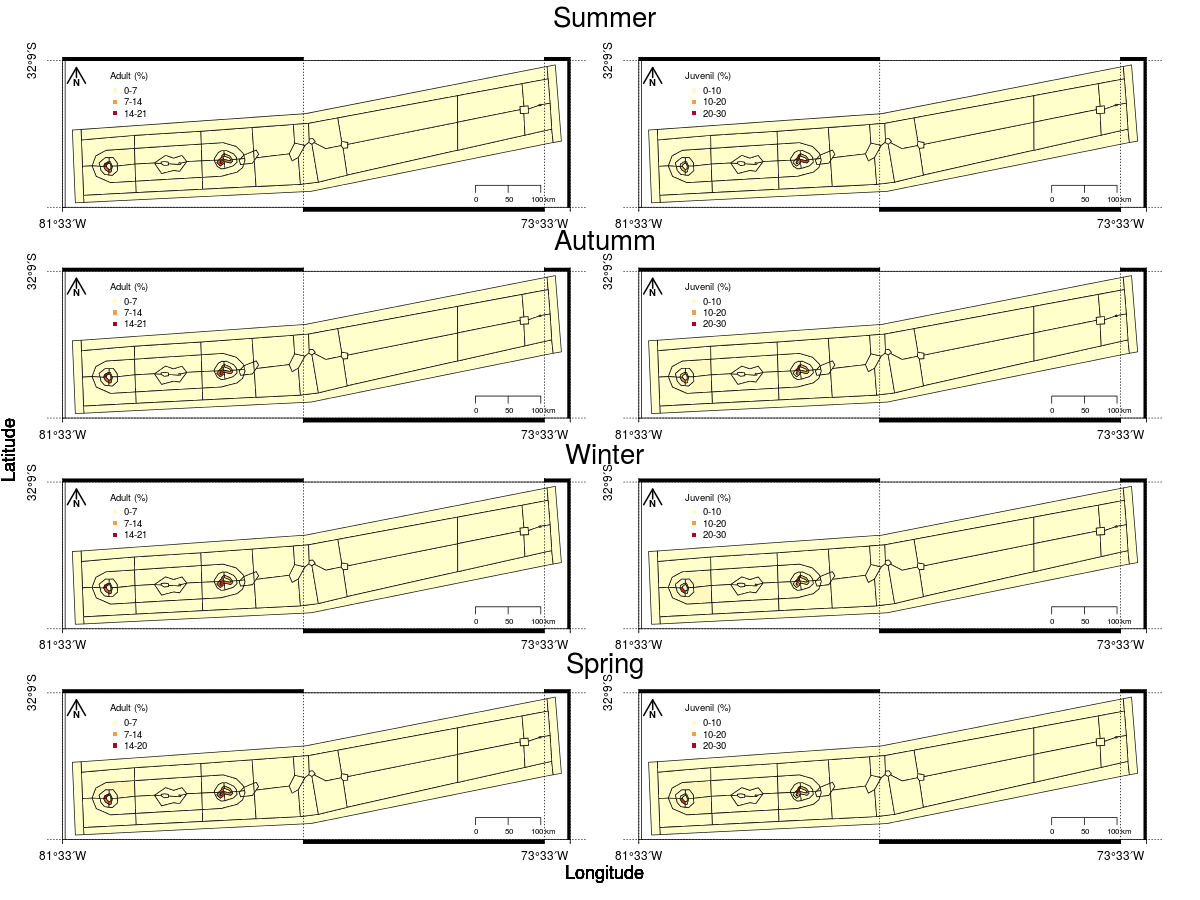

Supplement: S3 Fig — The ontogenetic stages—adults (Left column) and juveniles (right column)—were based the size at first maturity estimated by Ernst et al. 2016 [50]. (PNG) [file pone.0212485.s011.png]

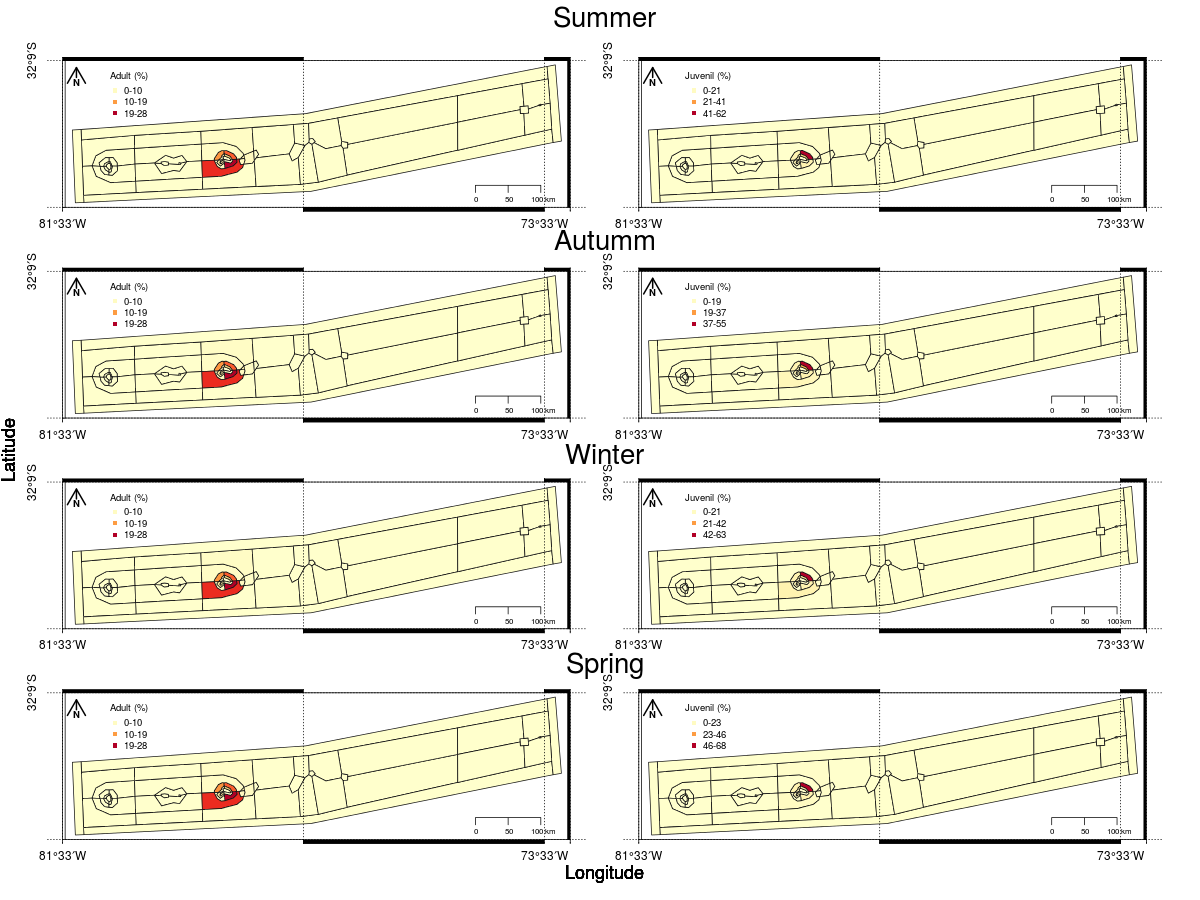

Supplement: S4 Fig — The ontogenetic stages—adults (Left column) and juveniles (right column)—were based on size at first maturity estimated by Guerrero and Arana 2009 [105]. (PNG) [file pone.0212485.s012.png]

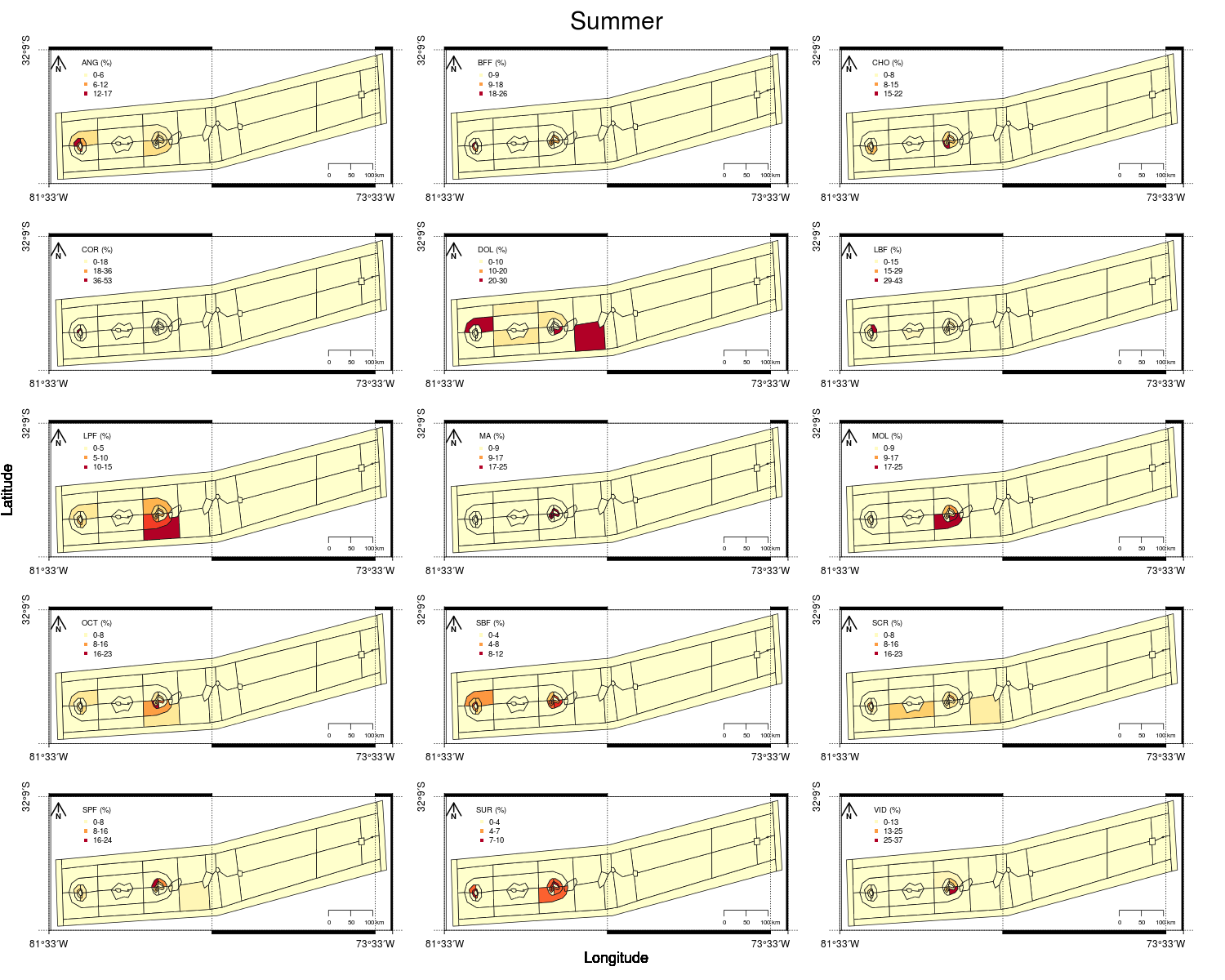

Supplement: S5 Fig — The functional group codes are as of S1 Table. (PNG) [file pone.0212485.s013.png]

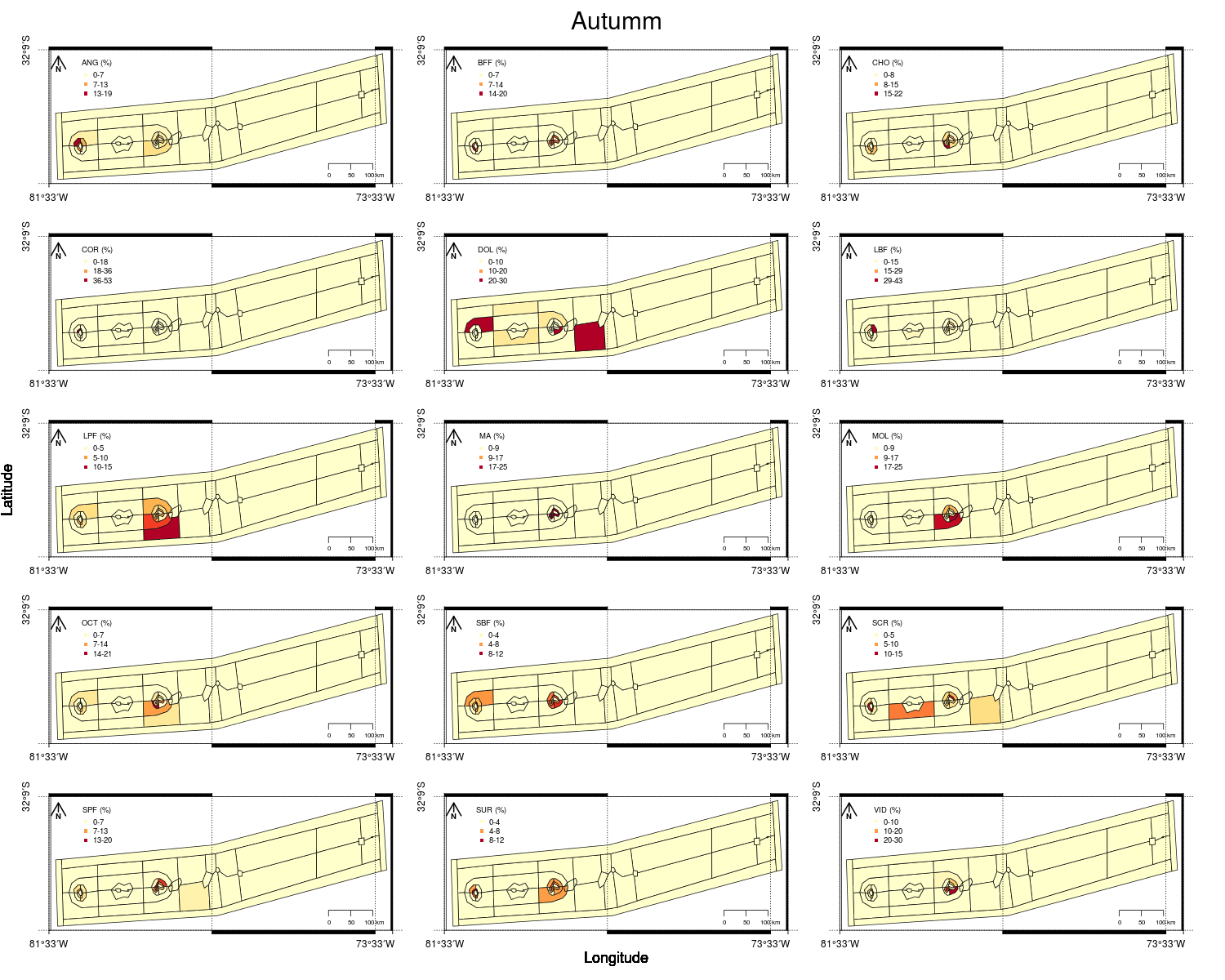

Supplement: S6 Fig — The functional group codes are as of S1 Table. (PNG) [file pone.0212485.s014.png]

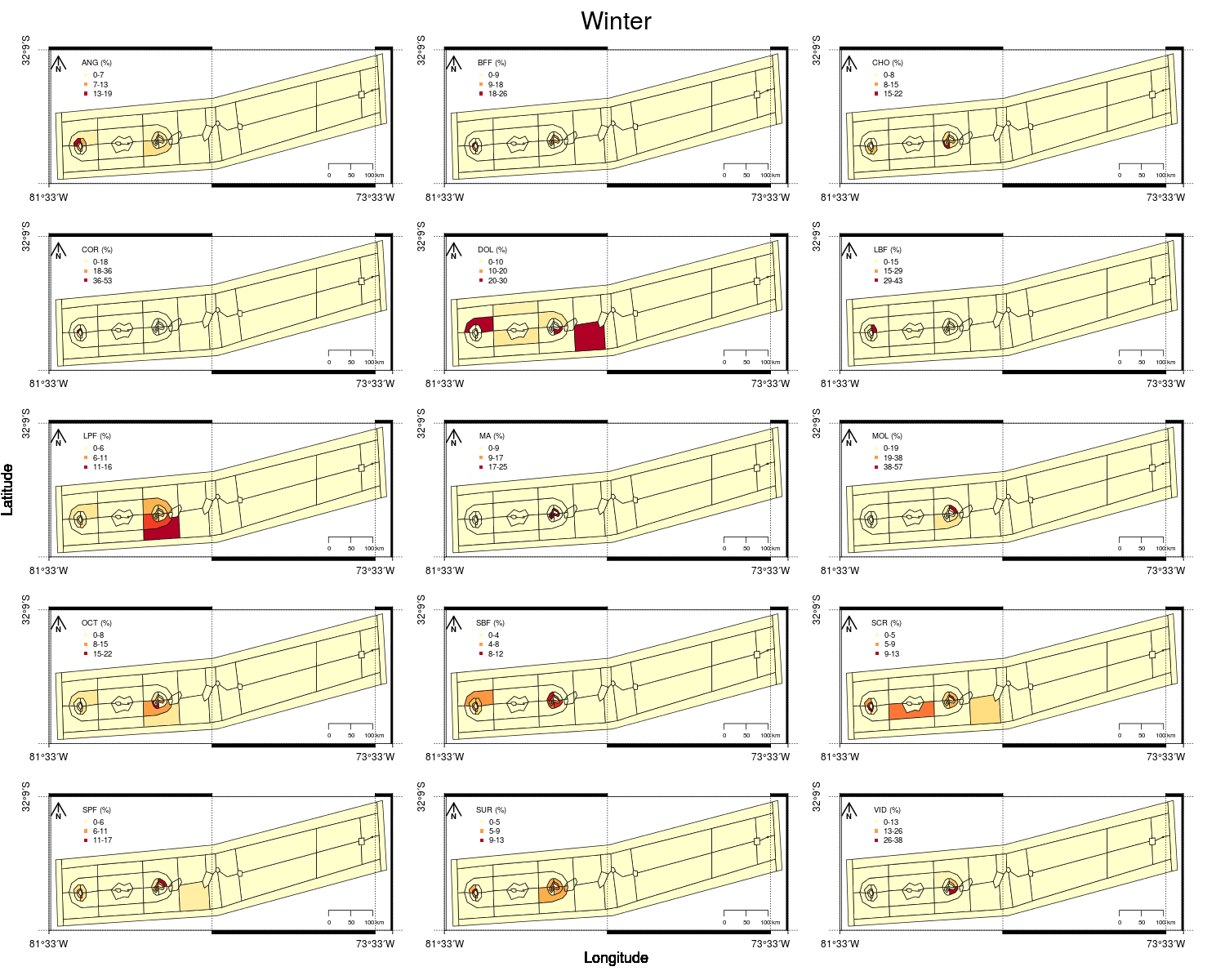

Supplement: S7 Fig — The functional group codes are as of S1 Table. (PNG) [file pone.0212485.s015.png]

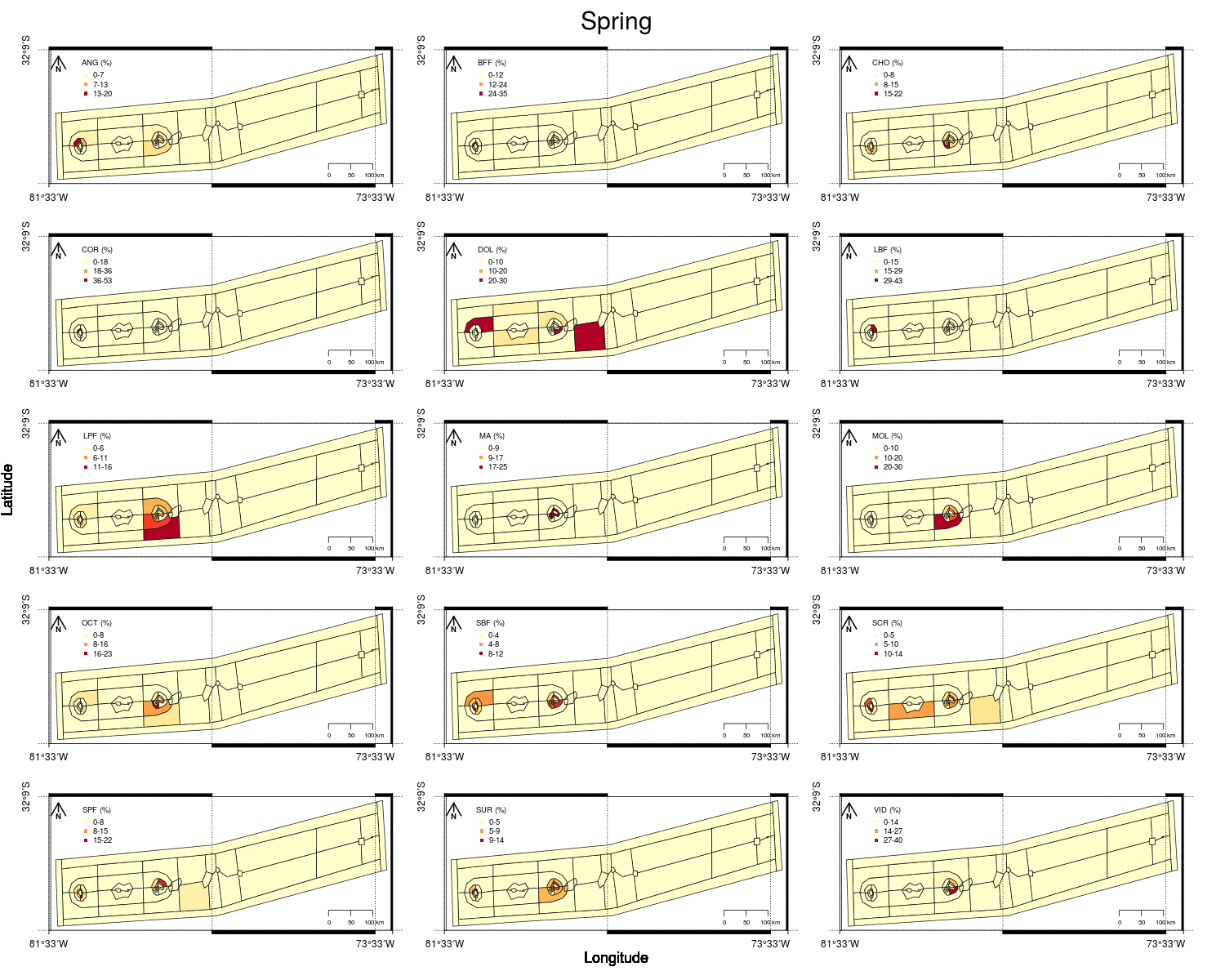

Supplement: S8 Fig — The functional group codes are as of S1 Table. (PNG) [file pone.0212485.s016.png]

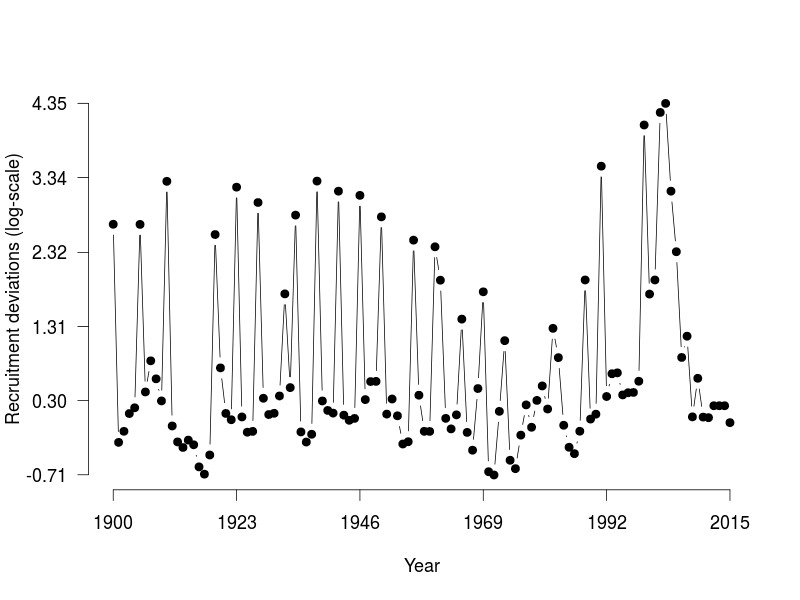

Supplement: S9 Fig — (PNG) [file pone.0212485.s017.png]

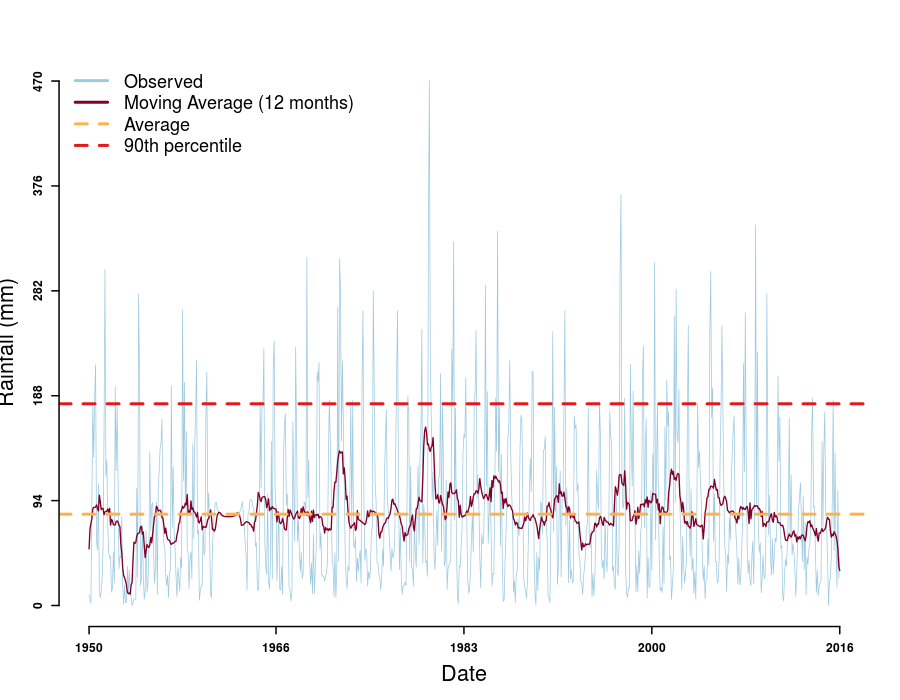

Supplement: S10 Fig — The 90th percentile represents the extreme rainfall events. (PNG) [file pone.0212485.s018.png]

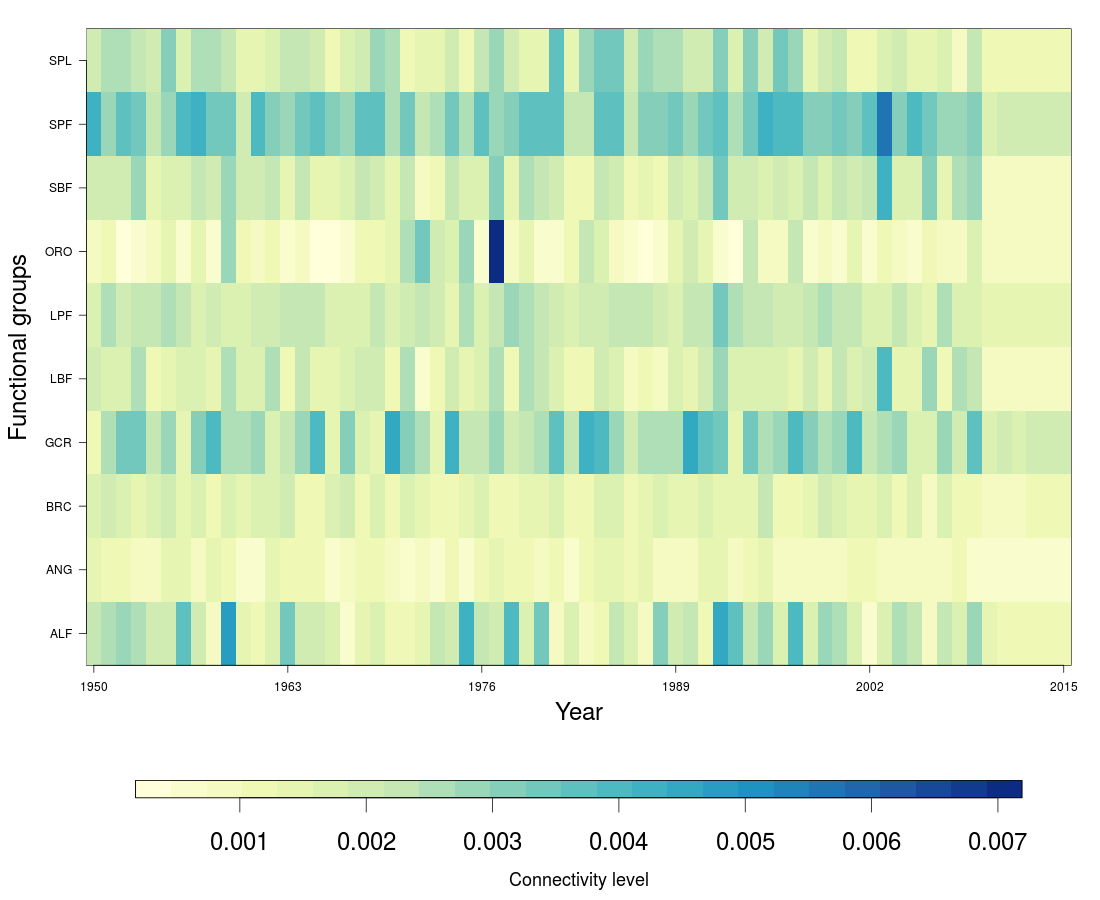

Supplement: S11 Fig — (PNG) [file pone.0212485.s019.png]

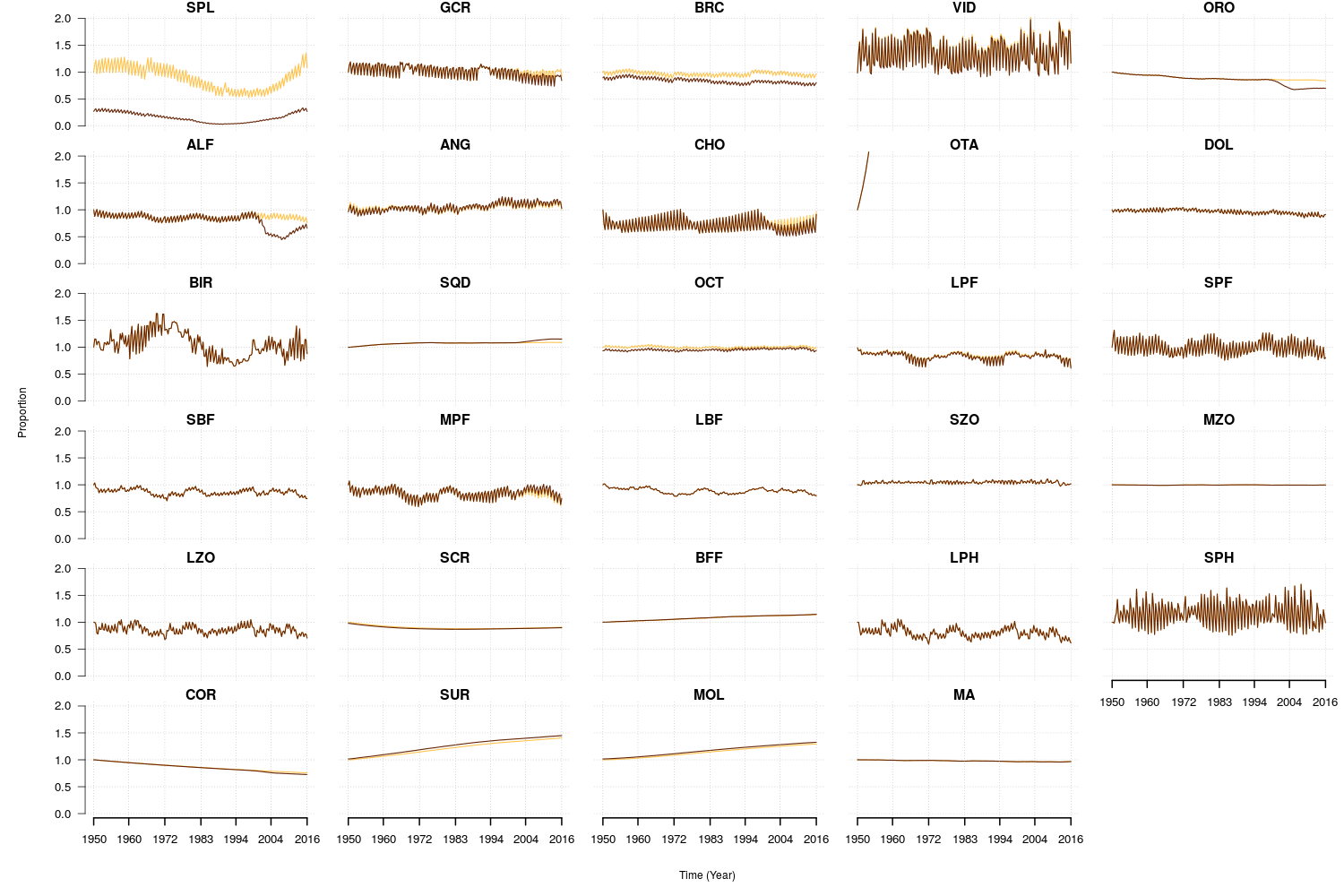

Supplement: S12 Fig — (PNG) [file pone.0212485.s020.png]

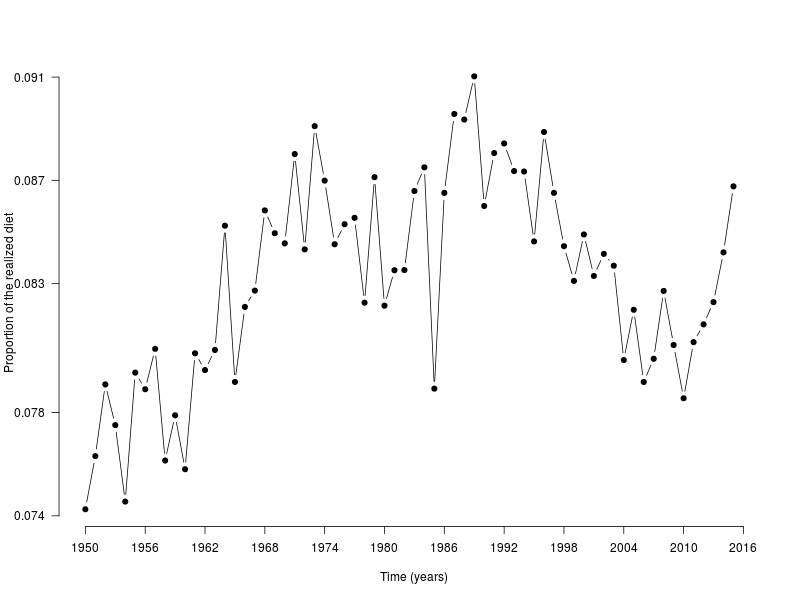

Supplement: S13 Fig — (PNG) [file pone.0212485.s021.png]

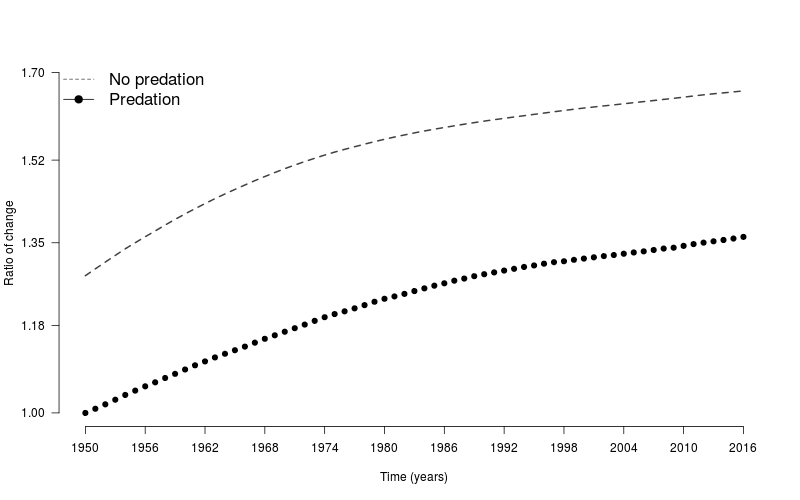

Supplement: S14 Fig — The black dots represent the relative biomass of sea urchin under the predation effect of moray eels. The dotted grey line represents the relative biomass of sea urchin without the predator effect of moray eels. Both time series are relative to the initial biomass of sea urchin. (PNG) [file pone.0212485.s022.png]
